# The Mechanism of Mineral Nucleation and Growth in a Mini-Ferritin

**DOI:** 10.1021/jacs.5c05464

**Published:** 2025-10-06

**Authors:** Colin C. Gauvin, Monika Tokmina-Lukaszewska, Hitesh Kumar Waghwani, Sterling C. McBee, Trevor Douglas, Brian Bothner, C. Martin Lawrence

**Affiliations:** † Department of Chemistry and Biochemistry, 33052 Montana State University, Bozeman, Montana 59717, United States; ‡ The Thermal Biology Institute, Montana State University, Bozeman, Montana 59717, United States; § Department of Chemistry, 1772 Indiana University, Bloomington, Bloomington, Indiana 47405, United States

## Abstract

Iron is an enigmatic element. While necessary for life, as Fe­(II) it also catalyzes formation of reactive oxygen species. To mitigate this, cellular life has evolved the ferritin protein superfamily, which includes the 24 subunit ferritins and bacterioferritins, and 12 subunit mini-ferritins (DPS). Each catalyze the oxidation of Fe­(II) to ferric oxyhydroxide, which is then sequestered within the hollow protein shell. While there is a wealth of structural information on unmineralized ferritins, high resolution information on iron loaded ferritins is lacking, and the mechanism of iron mineralization is poorly understood. To address this, we followed iron loading in a mini-ferritin by cryo-EM. We determined a 1.86 Å structure in the unmineralized state, as well as a 1.91 Å structure of an early, iron loading state in which the mini-ferritin catalyzes nucleation of ferric oxyhydroxide at the acidic 3-fold pores. Mechanistically, a conserved crucible of precisely positioned glutamates and unsaturated main chain carbonyls are employed as a template to catalyze nucleation. A 2.4 Å structure at a later time point was also determined, revealing the role of a second constellation of main-chain carbonyls on the interior surface that subsequently supports crystalline mineral growth, that then proceeds into the center of the particle. Notably, the visualized mineral is consistent with one of two competing structural descriptions for ferrihydrite. This study provides the first pseudoatomic level observation of controlled mineral nucleation and growth in any member of the ferritin superfamily, and informs general mechanisms of nucleation and biomineralization.

## Graphical abstract

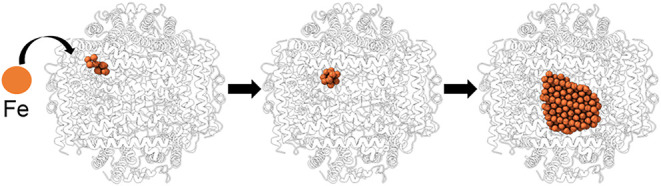

## Introduction

Iron is an essential element, playing a critical role in a wide variety of metabolic processes across all known life forms.^1–5^ However, free iron, in its ferrous form [Fe­(II)] is susceptible to oxidation by hydrogen peroxide, which results in generation of the highly toxic hydroxyl radical (OH·). Therefore, careful regulation of free Fe­(II) in cells is necessary to prevent the formation of this damaging free-radical.^1–3,6,7^

The ferritin protein superfamily, nearly ubiquitous throughout the tree of life, has evolved to address iron storage, iron toxicity and free radical formation.^2,8,9^ Ferritins oxidize Fe­(II) to Fe­(III) and store it within the ferritin nanoparticle as hydrated ferric oxyhydroxide. The ferritin superfamily is frequently recognized to consist of three major classes; (i) ferritin, (ii) bacterioferritin and (iii) DPS (DNA Protection in Starved cells).^8,10–13^ The eponymously named ferritin is a hollow nanocage 10–12 nm in diameter, composed of 24 subunits that assemble with octahedral (432) symmetry. The protomer is a 4-helix bundle that typically houses a binuclear iron center coordinated by glutamate and histidine residues. The metals are sequestered within the middle of the 4-helix bundle, and serve as the site of ferroxidase activity.^13–18^ Like ferritin, bacterioferritin utilizes a similar ferroxidase center (FOC) and 24 subunit quaternary structure, but incorporates a heme group at each of the 2-fold symmetric subunit interfaces. In contrast, while DPS also uses the 4-helix bundle, this “mini-ferritin” instead forms a smaller, dodecameric 9 nm nanocage with tetrahedral (23) symmetry.^14^ Further, rather than a ferroxidase center sequestered within the 4-helix bundle, the FOC is instead found on the inside of the shell at the intersubunit interface.^19^

Ferritin, bacterioferritin and DPS are each capable of oxidizing Fe­(II) to Fe­(III) via coupled reduction of O_2_ to H_2_O_2_, or H_2_O_2_ to H_2_O. At neutral pH, however, Fe­(III), is insoluble at concentrations above 10^–18^ M.^12^ Ferritins once again contribute a solution, storing Fe­(III) as mineralized ferric oxyhydroxide (Fe­(O)­OH) inside their hollow core.^10,20^ While the composition of the mineral varies, it is generally some form of ferrihydrite,^20–22^ whose structure is actively debated.^23–25^ The larger ferritins can store up to 2500–5000 mineralized iron atoms per particle, while the mini-ferritins can store as many as 500.

An additional member of the ferritin family, overlooked at times, is DPSL. First identified in hyperthermophilic archaea, DPSL forms a 12-subunit (dodecameric) nanocage that preferentially utilizes H_2_O_2_ as a substrate.^17,18,26,27^ For these reasons, DPSL was first perceived as a DPS-Like mini-ferritin. However, structural studies revealed a surprise; DPSL lacks the DPS ferroxidase site and instead retains a bacterioferritin-like ferroxidase center in the middle of the 4-helix bundle.^18^ For these reasons, DPSL is proposed to lie at the evolutionary interface between the mini- and maxi-ferritins.^18,28–30^ Importantly, DPSL further differentiates itself from both the (bacterio)­ferritins and DPS by the presence of two conserved cysteine residues that are juxtaposed between the ferroxidase center and the exterior surface of the particle. Together, the conserved cysteines and the bacterioferritin-like ferroxidase center serve as hallmarks of the archaeal DPSL proteins that are easily identified within the sequence as a “thioferritin” motif, and serve to differentiate DPSL as a distinct member of the ferritin superfamily.^28^ Whether the cysteine residues in the thioferritin motif are redox active, play a role in catalysis, or gate access to the bacterioferritin-like ferroxidase center is currently unknown. Importantly, however, DPSL proteins clearly participates in the oxidative stress response in anaerobic bacteria such as *Bacteroides fragilis*, the most commonly isolated bacteria from anaerobic infections.^28^ In this light, DPSLs are of clinical interest as well.^28^

While transmission electron micrographs of mineralized ferritin show well-defined nanoparticle crystallites encapsulated within the protein shell, and X-ray and electron powder diffraction studies suggest the mineral is present as crystalline ferrihydrite,^20,31^ efforts to follow the nucleation and mineralization reaction at higher resolution have proved challenging. Rather than mineralized iron, crystallographic studies frequently resolve only a handful of hydrated iron atoms per subunit on the interior surface the protein, or very small metalloclusters in the case of mammalian ferritins and the mini-ferritin *H. salinarum* DpsA. Mineralization of mammalian ferritins, which are hetero-oligomers of light (L) and heavy (H) chains, is thought to proceed with oxidation of Fe­(II) to Fe­(III) at the ferroxidase center in the H subunit, followed by transit of Fe­(III) through a 20 Å channel along the center of the 4-helix bundle to emerge into the interior cavity.^32^ Crystallographic studies then identify a tri-iron metallocluster, coordinated by a cluster of 3 glutamates in the L-ferritin B-helix (D60, E61, E64), as a putative nucleation site for mineralization.^33^ In contrast, the mini-ferritin from *Halobacterium salinarum* utilizes the conserved intersubunit DPS ferroxidase site to oxidize iron, followed by assembly of a 4-iron-3-oxo metallocluster coordinated by 3 symmetry related glutamate residues (E154) on helix D along the 3-fold axis.^19^ Although these small L-ferritin and DpsA metalloclusters are found at different locations in the particle and their structures are distinctly different from each other and the proposed structures for ferrihydrite, we find it interesting that they each utilize 3-fold (DpsA) or pseudo-3-fold (L-ferritin) symmetry to nucleate an initial metallocluster. But despite these insights, important details on molecular interactions that result in biomineral nucleation and growth remain unclear.^34^

We hypothesized such structures might be significantly more amenable to cryo-EM. Indeed, here we report not only the high-resolution (1.86 Å) unmineralized structure of DPSL from *Pyrococcus furiosus* (Pf-DPSL), but three additional structures as well; specifically, a nascent “nucleated” structure with low iron content, as well as mineralized structures with intermediate and higher iron content. These structures provide unique insight into the mechanisms of ferritin iron mineralization and storage across four distinct stages of the biomineralization process; (i) mineral free, (ii) initial nucleation, (iii) early deposition of the iron oxide core, and (iv) transition to the fully mineralized form.

## Experimental Section

### Expression and Purification of Pf-DPSL

Pf-DPSL was expressed and purified as previously described.^26^ However, a brief outline is provided in the Supporting Information.

### Iron Loading

For iron loading assays, protein was diluted to a final concentration of 0.2 mg/mL in 50 mM MOPS, 100 mM NaCl, pH 6.5 buffer. FeSO_4_ was dissolved into 0.1% HCl, and stoichiometric amounts were mixed with the diluted protein. Final concentrations were always calculated as the ratio of Fe^2+^ to protein dodecamer. For assays including hydrogen peroxide, ratios were 0.5 H_2_O_2_:1.0 FeSO_4_.

### Vitrification for cryo-TEM

Four μL of protein at 2 mg/mL protein was applied to either Quantifoil 300 Mesh Cu/Carbon R 1.2/1.3 or Au-flat 300 Mesh 1.2/1.3 (EMS AUFT313–50) grids. The samples were blotted stringently and plunge frozen into liquid ethane using a Vitrobot Mark IV (Thermo Scientific). Notably, in the absence of NaCl, Pf-DPSL exhibits a strong preference for carbon grid supports, while the addition of 100 mM NaCl (incorporated into the size exclusion buffer, see above) causes Pf-DPSL to partition into the ice, where at sufficient concentrations it may form close-packed arrays with a 9 nm particle spacing, reminiscent of the hexagonal lattices previously observed in DPS-DNA biocrystals (Figure S1).^12,35^

### Cryo-TEM Data Collection

The prepared grids were loaded into our home 200 kV Talos Arctica G2 (Thermo Scientific) and imaged with the K3 camera (Gatan). Data collections were performed using SerialEM (unmineralized, nucleated), and SmartScope (iron loaded), using a 5 × 5 multishot scheme, or beam-image-shift (BIS) distance of 7.5 μm, where appropriate.^36,37^ The unmineralized data were collected at 0.345 Å/pixel in super resolution mode. The nucleated and iron loaded data sets were collected at 0.69 Å/pixel, without using super resolution. The dose for all data sets was 55 e^–^/Å^2^. A defocus range of −0.6 to −1.5 μm was used for all data collections.

### Single Particle Analysis of Empty Pf-DPSL

The workflow for the single particle analysis of unmineralized Pf-DPSL is presented schematically in Figure S2. A total of 27,973 movies were recorded with subsequent patch motion and CTF correction using CryoSPARC Live.^38^ Movies were curated based on quality and resolution of the CTF fit, calculated defocus, total motion, and relative ice thickness, reducing the data set to 13,996. After data collection, 100 micrographs were picked using the CryoSPARC blob picker, using an 80–100 Å blob size. Particles were extracted, and 2D classification was performed in CryoSPARC. Particles were selected that contributed to 2D classes exhibiting visual secondary structure. Those particles were used to train a crYOLO model that was then used to pick particles in all remaining movies.^39^ Particles were extracted again and reclassified in 2D. Obvious junk classes were removed, and 200,000 particles were used to perform a 2 class *ab initio* reconstruction with enforced tetrahedral symmetry, generating a good map and a junk map. Heterogeneous refinement of all particles against the two maps was performed iteratively in CryoSPARC, each time selecting just the particles that aligned well to the good map for the next iteration. After three iterations, the remaining 2,583,454 particles were put into homogeneous refinement with enforced tetrahedral symmetry. The aligned particle stack then underwent two iterations of global CTF refinement in CryoSPARC, followed by local CTF refinement, before a final homogeneous refinement that enforced tetrahedral symmetry, giving a map with a resolution of 1.86 Å as judged by gold standard Fourier shell coefficient of 0.143.

An initial homology model was generated using Phyre2.^40^ The homology model was symmetry expanded onto the existing *Saccharolobus solfataricus* DPSL structure (PDB 2CLB) using ChimeraX,^18,41^ and docked into the experimental half maps using the Phenix module, emplace_local.^42,43^ The consensus map was then sharpened using Phenix b_iso_to_d_cut, and the docked model was iteratively rebuilt and refined against the sharpened map using Coot and Phenix real_space_refine.^42–44^ Metrics for model and map validation are presented in Table S1.

### Single Particle Analysis of Nucleated Pf-DPSL

Workflow for the single particle analysis of nucleated Pf-DPSL is presented schematically in Figure S3. The workflow was identical to the unloaded data set as far as the first model from homogeneous refinement. A total of 3915 exposures were recorded. 3094 of these exposures were selected based on quality metrics, with 1,742,467 particles going into the homogeneous refinement, giving an initial structure at 2.33 Å resolution by gold standard FSC. This map showed clear density in the 3-fold acidic pores corresponding to the presence of mineralized iron. The particles were then aligned to the D2 symmetry axes (*x*, *y*, *z*) and symmetry expanded by 222 point group symmetry, giving 4 virtual particles per real-particle, for a total of 6,969,868 virtual particles, each representing a unique 3-fold interface. A mask was placed around the 3-fold interface near the Glu50 residues on the *z* axis and focused 3D classification was performed, splitting particles into two classes based on the presence or absence of mineral within the masked region. Density was detected in the masked region for 3,584,523 virtual particles, indicating that roughly half the acidic 3-fold pores contained mineral. The symmetry expansion was then reversed for the mineral containing virtual particles, which reduced to 1,619,884 real mineral containing particles. The mineral containing real particles were then refined with tetrahedral point group symmetry, for a final structure at 1.91 Å by GSFSC. Model and map validation metrics are presented in Table S1.

### Single Particle Analysis of Iron Loaded Pf-DPSL

Workflow for the single particle analysis of iron loaded Pf-DPSL is presented schematically in Figure S4 and was identical to the unloaded data set as far as the first model from homogeneous refinement. A total of 13,577 exposures were recorded, with 9094 exposures selected based on quality metrics, and 3,153,592 particles for the initial map at 2.15 Å resolution. Protein density was then masked out, and CryoSPARC 3D Classification was used to sort particles based on the presence (1,504,967) or absence (1,650,473) of a strong mineral core at the particle center. Particles with mineral cores were then reconstructed using poses from the initial homogeneous refinement. The resolution of this reconstruction was 2.43 Å. Metrics for model and map validation are presented in Table S1.

### C3 reconstruction of Iron Loaded Pf-DPSL

D2 (222) is a symmetry subgroup of tetrahedral (T, 23) point group symmetry. However, the standard orientation for T symmetry in CryoSPARC is inconsistent with the standard orientation for D2. Thus, beginning with the 1,504,967 particles with a strong mineral core that were identified above, we used CryoSPARC’s Volume Alignment Tools to align the 2-fold particle axes with standard D2 symmetry (2-folds along *x*, *y*, *z* axes), to give a map in the D2 orientation. The D2 aligned particles were then D2 symmetry expanded and then realigned back to CryoSPARC’s T symmetry axes using the Align 3D Maps tool. Specifically, the D2 map (above) was aligned to a reference map in the tetrahedral orientation, and the symmetry expanded particle alignments were updated. This gave a D2 symmetry expanded data set of roughly 6 million particles in the original tetrahedral orientation (one 3-fold axis along z, 3 others running at oblique angles through the origin). A spherical mask 15 Å in diameter was then centered on the three Gly170 residues on the inner surface of the particle 3-fold along the *z*-axis (Mask A), and a binary 3D classification was performed, testing for the presence or absence of iron at site A. All mineral containing particles were retained, while those lacking mineral were discarded. Then, to exclude particles with mineral at two or more 3-folds, masks were placed sequentially at each of the remaining 3-fold positions, B, C and D. 3D classification was first performed with mask B, testing for the presence or absence of mineral at that 3-fold. Particles with mineral density at 3-fold B were then discarded, while those lacking mineral at B were retained (they have mineral at A). This was repeated with masks C and D, resulting in a final set of 395,423 particles with mineral at just a single 3-fold (mask A, on the *z* axis). Those particles were then reconstructed with C3 symmetry to generate a map with mineral growth from a single acidic 3-fold, at a final resolution of 2.4 Å. Workflow for this C3 reconstruction is presented schematically in Figure S5.

The protein structure was modeled as described above. To model the mineral, the ferrihydrite unit cell was expanded into a larger 2 × 2 × 2 supercell using Vesta, and then superpositioned upon the 4 well-ordered iron positions (see results).^24,45^ Iron and oxygens lying outside of the supporting density were then pruned from the structure until all remaining atoms were consistent with the density. The pruned model was then rigid body refined to optimize the fit to the density. In order to ensure the structure remained consistent with the ferrihydrite structure, individual atom positions were not refined. Metrics for model and map validation are presented in Table S1. Structural figures were prepared with ChimeraX, Pymol and the Caver 3.0 Plugin.^46–48^

### Strategies for Single Particle Analysis of Iron Loaded Ferritins

Additional thoughts on potential strategies for single particle analysis of iron loaded ferritins are presented in the Supporting Information.

### Native Mass Spectrometry

Iron loading was investigated using native mass spectrometry. The experiments were conducted on a SYNAPT G2-*Si* instrument (Waters) as described previously.^49,50^ Additional details are provided in the Supporting Methods.

## Results

### Structure of Pf-DPSL at 1.86 Å Resolution

Prompted by our earlier crystallographic work with DPSL from *S. solfataricus* (Ss-DPSL),^18^ we first screened Ss-DPSL for cryo-EM studies. However, initial single particle work gave maps with only modest resolution. For this reason, we turned to the *P. furiosus* protein (Pf-DPSL),^51^ for which we lacked structural information. We determined the 1.86 Å structure of Pf-DPSL, as purified from *Escherichia coli*, by single particle analysis using our in-house 200 kV Talos Arctica and Gatan K3 camera (Figures S1, S2 and S6). As expected, the structure revealed a dodecamer, with each of the 12 subunits housing a bacterioferritin-like ferroxidase center buried in the middle of a 4-helix bundle (Figure 1). The two iron atoms are coordinated by the conserved histidine, glutamate and aspartate residues of the thioferritin motif, while the conserved cysteines line the solvent channels leading from the exterior into the ferroxidase center (Figure S7). Further, like other mini-ferritins with 23 point group symmetry, opposite ends of the 3-fold axes are nonequivalent, giving rise to two classes of 3-fold pores, one rich in glutamate and/or aspartate residues (acidic or ferritin-like 3-fold pore), and a second, more hydrophobic pore (Figure 1). In Pf-DPSL the four hydrophobic 3-folds are constricted, while the four acidic or ferritin-like pores provide the largest openings into the interior of the particle.

**Figure 1:**
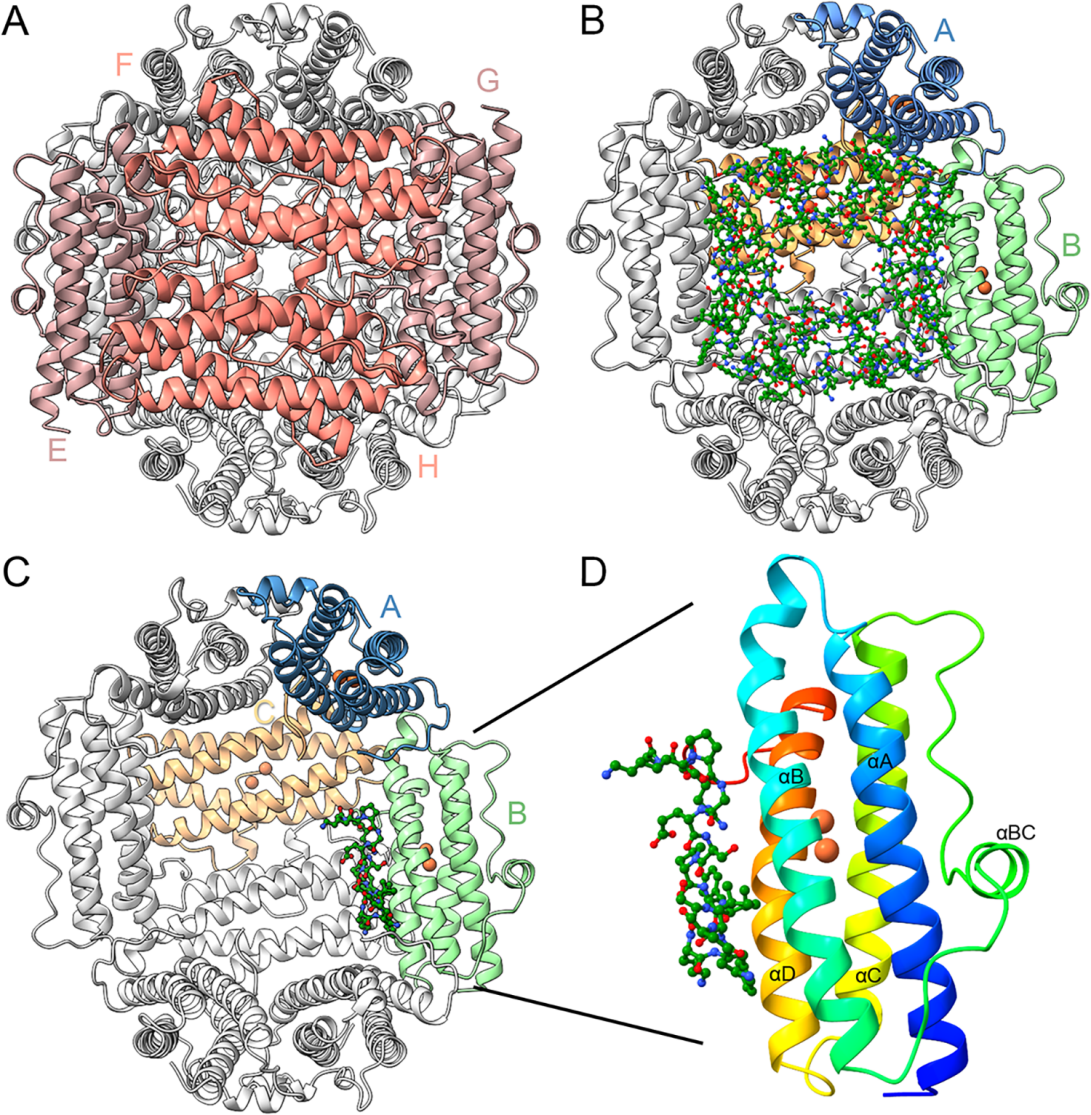
Structure of Pf-DPSL. (A) The dodecameric assembly viewed down the 2-fold axis (perpendicular to page). The 2-fold symmetric E and G subunits (left and right) are colored light pink, the 2-fold symmetric F and H subunits (above and below) in salmon. (B). The E, F, G and H subunits are removed, revealing the particle interior. The ordered C-terminal tails (ball and stick) contribute substantial structure to the interior of the nanoparticle (carbon in green, oxygen red, nitrogen blue). Chains A, B and C, which form one of the ferritin-like pores, are indicated in blue, light-green and sandy brown. (C). The C-terminal tail of just the B subunit is depicted, showing the relative orientation and contribution of a single protomer. (D) Subunit B is extracted and colored in Jone’s rainbow, with αA in blue, αB in cyan/green, αC in yellow, αD in orange/red, and the ordered C-terminal tail in green ball and stick. The small BC helix in the extension connecting helices B and C is also labeled (αBC). The two irons in the ferroxidase center, buried within the 4-helix bundle, are depicted as rust-colored spheres. The C-terminal tail runs parallel to αD toward the bottom of the page, showing unsaturated, solvent exposed main chain carbonyls.

### Ordered C-Terminal Tail

To our knowledge, reported DPSL structures are limited to the archaeal *DPSL* from *S. solfataricus*, and the bacterial DPSL from *B. fragilis*.^18,28^ While the structures are largely similar, the C-terminal tail in the bacterial structure remains on the exterior surface of the particle. In contrast, in the archaeal structure the C-terminal tail transits the protein shell to the interior of the particle, with the remaining residues disordered within the cavity.^18,28^ As expected, structural superposition shows that Pf-DPSL is more similar to its archaeal ortholog (Ss-DPSL Cα RMSD = 0.69 Å) than its bacterial cousin (Bf-DPSL, Cα RMSD = 2.72 Å), with the C-terminal tail also transiting into the interior of the particle. However, in contrast to the Ss-DPSL structure, as it emerges near the acidic 3-fold pore, we see 13 additional ordered residues (Gly170-Lys183, Figure 1).

The first three residues of the tail extend toward the center of the particle to Pro172, where the tail then turns sharply and takes a convoluted path, roughly parallel to the four-helix bundle, along the inside of the shell. Residues 179–181 then form a short 3/10 helix that anchors the C-terminal end of the tail, with Lys180 in a salt bridge to Asp139, Phe181 packed in a hydrophobic pocket against Leu140, and an ion-dipole interaction between the Glu147 side chain and the main chain amines of Val178 and Tyr174. In addition, sharp turns at Pro172 and Pro176 are each stabilized by intrastrand main chain hydrogen bonds.

### Unsaturated Protein Carbonyl Groups

Interestingly, this ordered structure leaves at least 9 unsaturated protein main chain carbonyl groups (Arg169, Gly170, Pro172, Pro176, Tyr177, Ser179, Phe181, Leu182 and Lys183) that project into the interior solvent. Multiplied by the 12-fold symmetry of the particle, the C-terminal tails contribute 108 unsaturated carbonyls to the interior surface of the particle, where they might potentially interact via ion-dipole interactions with Fe­(III) or participate in hydrogen bonds to the hydroxyl groups of mineralized ferric oxyhydroxide. With the addition of these ordered residues at the C-terminus, the measured interior diameter is approximately 30 Å.

### Nucleated DPSL

Ferritin, bacterioferritin and DPS can each utilize O_2_ or H_2_O_2_, although the (bacterio)­ferritins generally prefer O_2_, while DPS prefers H_2_O_2_. Similar to DPS, previous studies on Ss-DPSL and Pf-DPSL also show a preference for H_2_O_2_.^17,26^ Because the structure of a partially mineralized particle could provide mechanistic insight into the nucleation and initial deposition of iron oxyhydroxide, we pursued the structure of this potentially informative intermediate by cryo-EM. Following incubation with 50 iron atoms per cage and stoichiometric amounts of H_2_O_2_ for 10 min, native mass spectrometry on intact protein cages indicated an average mass of 257 032 Da (Figure 2). Relative to the unloaded particles (256 118 Da), this was a mean increase of 913.6 Da, corresponding to approximately 10 Fe­(O)­OH units per cage. However, the peak width of each charge state was clearly broadened relative to the unloaded particles, indicating a distribution of loading states. Indeed, when inflection points on each side of the most dominant charge state (35+) were used to calculate masses, lower and upper mass increases of 308.3 Da (∼3 Fe­(O)­OH units) and 1923.8 Da (∼22 Fe­(O)­OH units) were found, giving greater insight into the distribution of Fe­(O)­OH loading.

**Figure 2:**
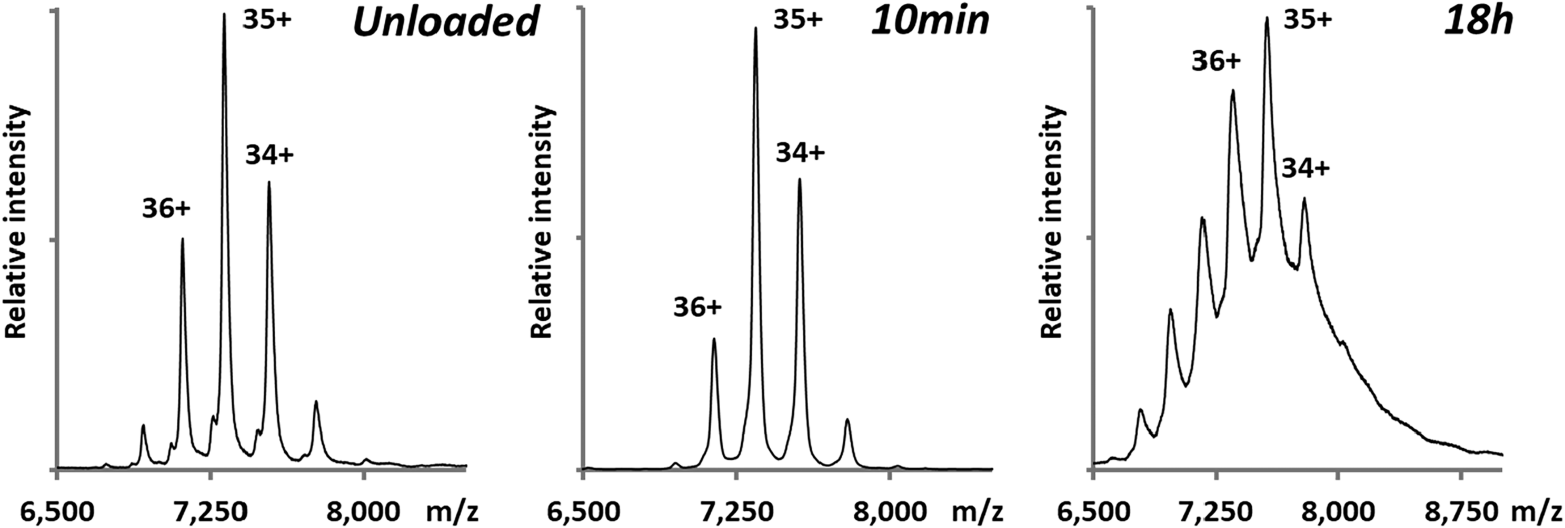
Native mass spectrum of unloaded (left panel),10 min Fe-loading in the presence of 50 Fe atoms and H_2_O_2_ (middle panel), and18-h Fe-loading in the presence of 500 Fe atoms and O_2_ (right panel). The unloaded, 10 min and 18-h Fe-loading reactions ran at molecular masses of 256,118.4 ± 19.6 Da, 257,032.3 ± 18.0 Da and 264,866.1 ± 18.4 Da, respectively. These masses represent the most dominant population in the Pf-DPSL particle ensemble. The dominant charge state was 35^+^ under all conditions.

The iron loaded particles were also vitrified on cryo-EM grids and imaged for single particle analysis (Figures S1 and S3). With the exception of 3000 particles, which were excluded from the reconstruction, particles exhibiting iron density in the core were not visible in the micrographs, nor upon 2D classification. Ultimately, a total of 1,742,467 particles were used in homogeneous refinement, yielding a map at 2.20 Å resolution that was largely similar to that for the unmineralized protein. However, clear density at each of the 3-fold acidic or ferritin-like pores suggested partial iron occupancies at this position. For this reason, the particle set was D2 symmetry expanded to give ∼ 7,000,000 virtual particles with unique 3-fold pores. A mask centered on the 3-fold *z*-axis that extended toward the center of the particle was then placed, and the virtual particles were subjected to 3D classification, giving two classes of roughly equal size, corresponding to iron deficient (3,385,345 virtual particles) and iron rich pores (3,584,523 virtual particles).

Unfortunately, an asymmetric (C1) reconstruction of the iron rich 3-fold particles, with or without homogeneous refinement, failed to resolve a high-resolution structure. Therefore, the symmetry expansion on the iron rich data set was reversed, giving ∼1,600,000 sorted particles with iron present in at least one 3-fold pore. Notably, with 3.6 million iron rich 3-fold pores from 1.6 million particles, this suggests an average of 2.25 iron rich pores per particle. These iron rich particles were then used for homogeneous tetrahedral refinement. With subsequent particle polishing, this gave a 1.96 Å structure. And despite averaging over the tetrahedral symmetry and the corresponding decrease in occupancy, this resulted in nicely resolved density for the iron atoms, and indicated the presence of ordered oxygen atoms as well (Figure 3).

**Figure 3:**
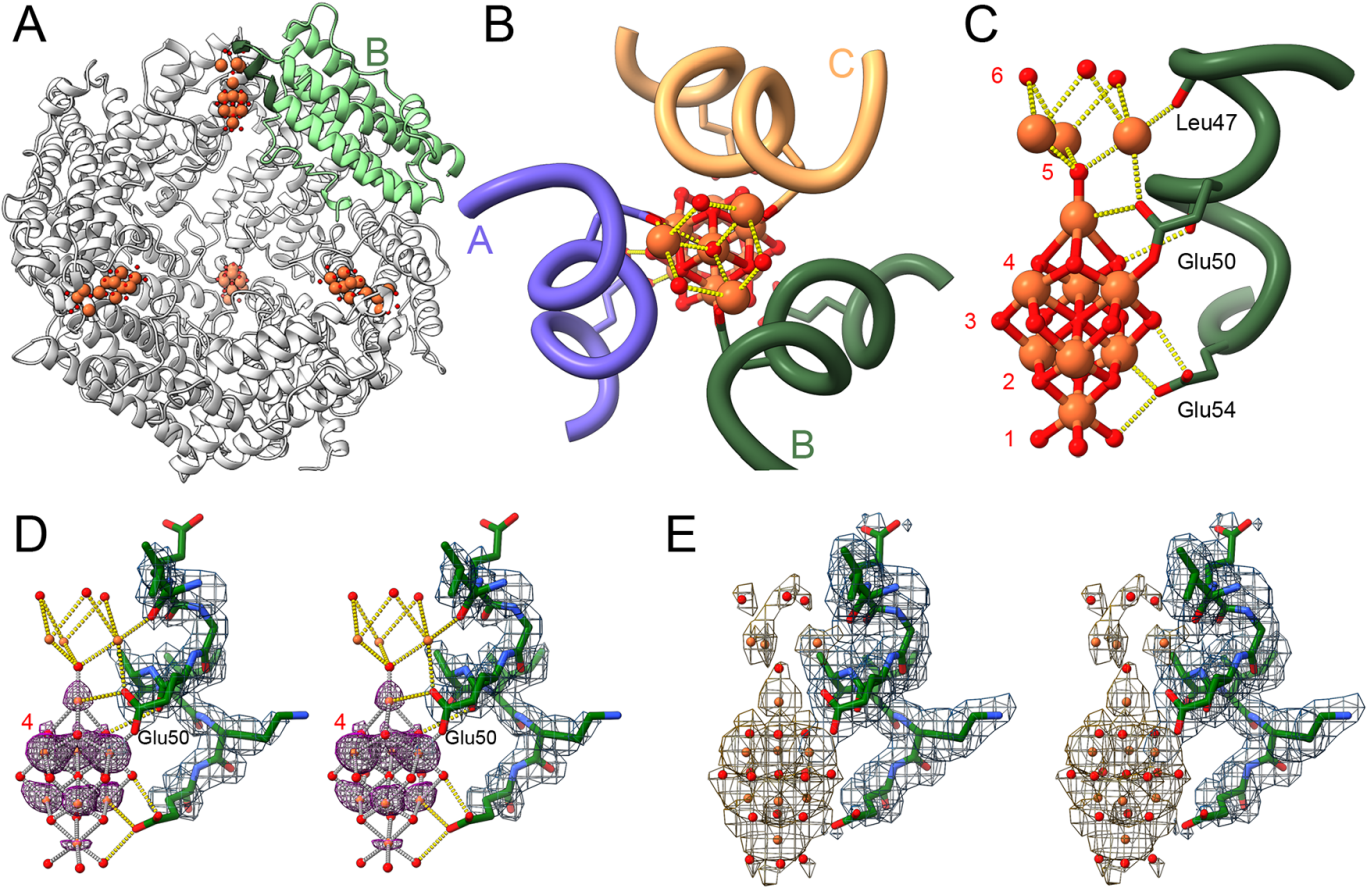
(A) Pf-DPSL dodecamer showing the location of the iron mineral at the four acidic pores. Iron atoms are rust-colored, oxygen atoms are red. The A, B and C subunits lie at the top of the structure, surrounding the vertical 3-fold axis and the topmost iron oxyhydroxide cluster. Additional 3-fold axes pass through the remaining iron oxyhydroxide clusters. The B subunit is depicted in light green. The N-terminus of the B helix (αB, Leu47-Glu54) is shaded dark green. (B) A view of the acidic pore looking from the exterior of the protein cage into the interior. The A, B and C subunits are in blue, green and sandy brown, respectively. Interacting side chains and carbonyl groups are displayed. (C) A “side” view of the nucleated mineral within the acidic pore. Relative to panel B, the image is rotated 90° about the horizontal axis, with only the B subunit (green) depicted for clarity. The exterior surface of the cage is at the top of the mineral, and the interior of the cage is as the bottom of the mineral. The Leu47 carbonyl lies at the top, Glu50 carbonyl and side chain in the middle, and Glu54 at the bottom. Numbered oxygen layers are indicated with red numerals. (D) Stereo “side” view with the B subunit in green sticks, and the iron and oxygen atoms of the nucleated mineral as orange and red spheres. Bond distances less than 2.2 Å are shown in silver dashes, the remainder in gold. Potential density for the mineral and protein is in magenta and “Dodger” blue, respectively, each contoured at 7.0 σ. At this level there is strong density for the protein main chain and for the bottom 8 irons in the mineral. Density is also present for the 3 oxygens in layer 4, adjacent to Glu50. (E) As in panel D, but the contour level adjusted to 2.5 σ for both protein and mineral, with the mineral isonet in beige, and the dashed bonds between Fe and O atoms removed for clarity. At 2.5 σ the isonet encloses the entire mineral model and most of the protein side chains. At intermediate contour levels, density is also suggestive of the additional mineral oxygens.

### Nucleated Structure

The overall structure of the iron nucleated particle is largely similar to the unmineralized structure, with a Cα RMSD of 0.37 Å. However, within the pore, we were able to model 11 iron atoms and 19 oxygen atoms in a 3-fold symmetric structure (Figure 3). Iron oxide and iron oxyhydroxide mineral structures are commonly described as planes of close packed oxygen atoms, with iron atoms present at various interstitial positions between the planes. Examples include the hexagonal close packed (ABABAB) structure of hematite, the cubic close-packed structure of maghemite (ABCABC), as well as the two competing models for ferrihydrite.^23,24^ In this context, the locations of oxygen atoms within the 3-fold pore are indeed consistent with planar close-packed structures. Moving along the 3-fold axis from the interior of the particle outward (bottom to top in Figure 3), the six oxygen layers are stacked in an ABCACA manner, with the four innermost layers (ABCA···) resembling a cubic close-packed structure (ccp), while the 4 outer layers (..CACA) are instead hexagonal close-packed (hcp) (Figure S8). In this model, the interface between the two middle layers (..CA..) represents an overlap between the two packing modes, i.e., a stacking fault.

The number of oxygens in each layer is quite small. Each of the A and B layers has only 3 oxygens, symmetrically arranged about the 3-fold axis, while the C layers have a single oxygen atom coincident with the 3-fold, which in the case of the third layer, is surrounded by six additional in-plane oxygens. Iron is present at each of the interstitial vacancies between the oxygen planes, giving a close packed iron atom lattice as well. The eight iron atoms in the innermost 4 layers show clear octahedral coordination to six oxygens, while the last 3 irons at the top of the structure, although ordered, appear under-coordinated. Measuring between oxygens in the top and bottom layers, the mineralized iron structure extends nearly 15 Å along the 3-fold axis, but is only 6 Å at its widest point (O to O across layer 3). Many of the oxygen atoms on the surface of the mineralized structure also appear undercoordinated, with only 2 or 3 bonds to Fe, suggesting they are present as hydroxyl moieties, rather than O^2–^. This is consistent with the expected iron oxyhydroxide nanomineral, as are the observed Fe–O bond distances, which range from 1.95 to 2.15 Å for the 17 oxygens in the bottom 5 layers. The outermost layer of 3 ″oxygens″ (panel 3C, top) is an exception, where average Fe–O bond distances are 2.54 Å, suggesting these might instead be strongly ordered waters.

### Protein-Mineral Interactions I

Residues in the 3-fold channel that interact with the mineralized iron include Leu47, Glu50 and Glu54 (Figure 3C). The role of Leu47 is the most straightforward, where the main chain carbonyl coordinates iron atoms in the exterior iron layer. In contrast to the ionic interactions between the iron and the mineralized oxygen atoms, at 2.5 Å the carbonyl oxygen–iron bond distance is significantly greater.

### Glutamate 50

The role of Glu50 is more complex, as the negative charge appears to be delocalized over both side chain oxygens, allowing interactions with 3 different iron atoms. The strongest interaction is with iron in the middle, or third iron layer of the complex, where the Oε_1_-Fe bond distance is 2.0 Å. In addition, Oε_2_ lies 2.9 Å from the single iron in layer 4, and 3.0 Å from the nearest iron in layer 5. Importantly, Glu50 also contributes its main chain carbonyl group. But unlike the Leu47 carbonyl, the Glu50 carbonyl does not interact with an iron atom, but instead lies within hydrogen boding distance (3.1 Å) of an oxygen atom in layer 4. This suggests this specific oxygen is present as hydroxide, which is consistent with the expected oxyhydroxide nanomineral.

### Glutamate 54

Both oxygens in the Glu54 side chain also interact with the mineral. As might be expected, Oε_1_ again interacts with iron, with a 2.65 Å bond distance to the nearest iron in layer 2. Unlike Glu50, however, Oε_1_ also interacts with oxygen in the bottommost layer, and O_ε2_ is 2.45 Å from oxygen in the third layer, suggesting hydroxide in the first and third oxygen layers as well. Thus, while the protein carboxylate and carbonyl moieties present within the “acidic” pore accommodate the positively charged iron, a second major theme is the use of these carboxylate and carbonyl oxygens to coordinate surface hydroxides. For these reasons, inorganic oxygens with fewer than 4 iron bonds were modeled as hydroxide ions.

### The Nucleation Site is Preformed

Leu47 and Glu50 are present at the N-terminus of the B helix (αB), defined as residues 46–76 by the Kabsch and Sander algorithm of DSSP,^52^ in both the unmineralized and nucleated structures. With ideal helical geometry, the carbonyl groups in these residues would be expected to hydrogen bond with main chain NH groups at residue i+4. DSSP also calculates electrostatic energy values for each putative hydrogen bond in a structure, with typical values for helical residues ranging between −2.0 and −3.0 kcal/mol. Because DSSP ignores the presence of iron, for the iron nucleated structure it calculates energies of −0.5 kcal/mol for both Leu47 and Glu50. Consistent with these low energy values, the carbonyls for Leu47 and Glu50 point slightly away from the helix. Accordingly, rather than an optimal H-bond distance of 2.8 Å, distances to the corresponding main chain i+4 NH groups are 3.8 and 4.2 Å for the Leu47 and Glu50 carbonyls, respectively.

Importantly, when we examine the high resolution unmineralized structure, we find even greater distances between the main chain carbonyl and NH moieties (4.3 and 4.6 Å respectively), and DSSP again calculates H-bond energies of only −0.5 kcals/mol. This suggest that the carbonyl elements of this iron binding site are preformed. Indeed, both the unmineralized and iron nucleated structures each show a bend or kink at the N-terminal end of the B helix at Ala51 (Figures. [Fig fig1]C and 3A,C). This structural kink thus serves an important functional role; cocking the helix at this position exposes the carbonyl groups of Leu47 and Glu50 for interaction with the elements of the mineralization reaction. However, in contrast to these prepositioned main chain carbonyl groups, the carboxylate side chains of Glu50 and Glu54 are rotated away from the nucleation site in the unmineralized structure, implying subsequent movement of these side chains into the nucleated conformation as the reaction proceeds.

Several additional structural features are also noteworthy in the nucleated DPSL structure. In particular C-terminal residues Lys162 to Pro172, which transit the protein shell, show a minor rearrangement. This in turn appears to lock Trp154, which is poorly ordered in the unloaded particle, into a single, highly ordered side chain conformation (Figure S10). Trp154.serves as a backstop to His151 in the ferroxidase center. At the same time, it is only 12 Å away from the nucleated mineral on the opposite side, and is thus juxtaposed between the ferroxidase center and the nucleated mineral.

### Ferrihydrite

While the outermost layer of iron and oxygen exhibit extended bond distances and less regular geometry, the inner 5 oxygen layers and intervening iron atoms have a notably crystalline appearance. In this light, ferritin and DPS each mineralize iron as ferrihydrite,^20,22,53^ suggesting Pf-DPSL should as well. Currently, two different models for ferrihydrite are debated in the literature,^23,24^ although a recent *ab initio* thermodynamics study suggests they might both exist.^25^ Regardless, each model is in a hexagonal space group that includes a crystallographic 3-fold axis. For this reason, we aligned the 3-fold axis of each ferrihydrite model with the 3-fold axis in Pf-DPSL to compare these models to the nucleated DPSL structure. While the nucleated structure is not a perfect match to either ferrihydrite model, we note greater similarity to the model of Michel et al.^24^

In the Michel model, 4/5ths of the iron atoms are octahedrally coordinated with the remainder in tetrahedral coordination.^53^ Within the unit cell, this gives rise to one cluster of 4 octahedrally coordinated irons sitting on each of the 3-fold axes, with a fifth tetrahedrally coordinated iron immediately above (or below) them (Figure 4). We find similar 3-fold symmetric, 4-iron clusters in the nucleated DPSL structure. Specifically, the 4-iron cluster and associated oxygens in ferrihydrite superposition on the bottom 4 irons and surrounding oxygens that are coordinated by Glu54.

**Figure 4:**
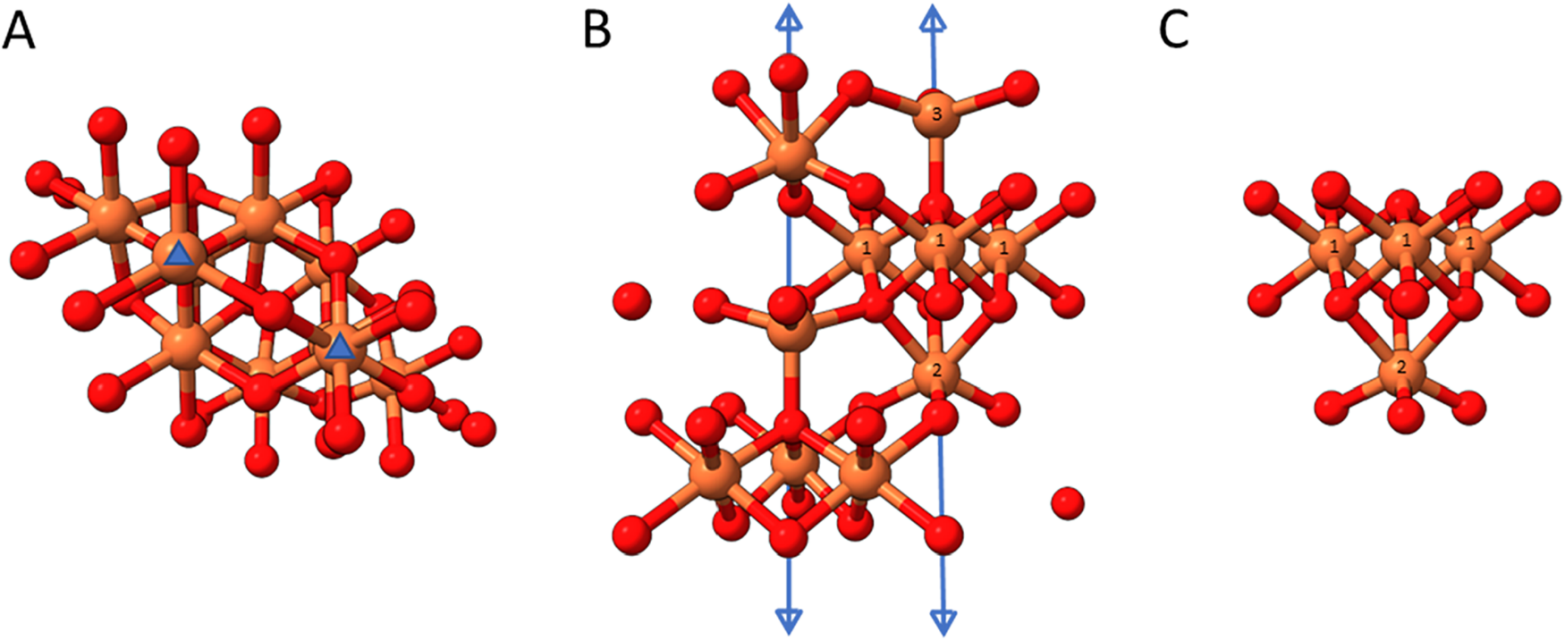
Ferrihydrite. (A) One unit cell of the ferrihydrite structure of Michel et al. (space group *P*6_3_mc) looking down c (*z* axis). Iron atoms are rust, oxygen atoms red. The position of the 3-fold symmetry elements are indicated with blue triangles. (B) The same structure, but looking along a*, perpendicular to the *z* axis. The 3-fold rotation axes are indicated by the extended triple headed arrows. Iron atoms on the right side of the unit cell are labeled in roman numbers (1,2,3) corresponding to Fe1, Fe2 and Fe3 atoms within the ferrihydrite asymmetric unit. While not indicated, a 2_1_ screw axis runs vertically between the two three folds. Fe1 and Fe2 are octahedrally coordinated, Fe3 shows tetrahedral coordination. (C) The same orientation as panel B, but isolating a single cluster of 4 octahedrally coordinated iron atoms. The 3-fold rotation axis runs vertically through the center of the cluster. The bottom Fe atom (Fe2) sits directly on the 3-fold axis, while the 3-fold passes between the top 3 iron atoms (Fe1), where the center iron is in front of plane of the page, and the other two are behind the plane of the page. This structure is highly similar to the structure of the bottom 4 irons in the nascent nucleation center in Pf-DPSL (Figure 3C).

When rotated 180° about the horizontal axis, the same cluster also superpositions on the central 4 irons coordinated by Glu50, although the top iron on the 3-fold (Fe2 in ferrihydrite) lacks obvious octahedral coordination. The two 4-iron clusters in the nucleated DPSL mineral thus share a central plane of hexagonally arranged oxygens (layer 3). Notably, the central oxygen in this plane is not well resolved. While this might be due to Fourier ripples from the six surrounding irons, we note an oxygen at this position would require octahedral coordination, as opposed to the tetrahedral coordination seen for the remaining oxygens, and the iron–oxygen bond lengths would be substantially longer (2.5 Å).

### Nucleation Mechanism

Overall, we see that DPSL utilizes a preformed, 3-fold symmetric crucible that is rich in precisely positioned carboxylate side chains and unsaturated main chain carbonyls. These moieties serve to template nucleation of the iron oxyhydroxide core, resulting in the construction of a nascent, 3-fold symmetric iron oxyhydroxide structure whose bottom most 4-iron cluster superpositions well on the ferrihydrite structure. In this context, we conclude that the 3-fold symmetry of DPSL is not a casual consequence of forming of a hollow dodecameric cage. Instead, the 3-fold symmetry of the quaternary structure is functionally important in catalyzing iron oxyhydroxide nucleation within the nanoparticle.

### Iron Loaded Structure

High resolution 3D visualization of the mineralized core of a ferritin or mini-ferritin particle has been an elusive goal for decades. A prerequisite for this is that the core itself must be highly ordered, preferably crystalline. To this end, we turned to an overnight incubation with ambient O_2_ in order to slow growth of the mineral core, hopefully providing a more ordered mineralized core.^20,54,55^ Native mass spectrometry of this sample indicated an average mass of 264,866.1 ± 18.4 Da (Figure 2C). Relative to the unloaded particles (256 118 Da), this was a mean increase of 8748 Da, corresponding to approximately 98 Fe­(O)­OH (∼89 g/mol) units per cage. However, the width of each charge state was greatly broadened relative to the unloaded particles, again indicating a distribution of loading states. When inflection points on each side of the most dominant charge state (35+) were again used to calculate masses, lower and upper mass increases of 7357 Da (∼83 Fe­(O)­OH per particle) and 10 864 Da (∼122 Fe­(O)­OH per particle) were found, giving insight into the distribution of Fe­(O)­OH loading. Because the peaks in the spectrum occlude each other, the actual distribution is probably wider than this.

These iron loaded particles were also vitrified for single particle analysis. Visual inspection of the micrographs and 2D classification revealed relatively heavy iron density in the center of roughly half the particles, with the remaining particles exhibiting a spectrum of decreasing iron densities, in which the smaller iron densities were no longer centered. We proceeded through initial homogeneous refinement without discriminating between particles, and then used a mask at the center of the nanocavity for 3D classification to select a subset of 1,503,830 iron loaded particles (Figure S4). Using poses from the initial homogeneous refinement, these most heavily iron loaded structures were further refined with tetrahedral averaging to give a final map at 2.4 Å resolution. Importantly, ferrihydrite does not share the DPSL 23 point group symmetry. Thus, even if the mineralized iron is sufficiently ordered, the tetrahedral averaging is expected to scramble, rather than resolve the structure, as it will incorrectly average across the ferrihydrite grain boundaries, both within and between particles. It is also expected to distribute density across the interior of the particle. Indeed, while the protein was well resolved, we were unable to model the mineral core itself. However, analysis of this iron loaded structure still provided important insight.

### Role for the C-terminal Tail

As expected, the most obvious ultrastructural change is the appearance of a large, dense, roughly spherical core, approximately 30 Å in diameter that fills the interior cavity of the particle (Figure 5). Aside from this, the structure of the protein remains largely similar to the nucleated form. This includes the C-terminal tail, which remains ordered along the interior surface of the particle, where it appears to interact strongly with the mineral core. From the mechanistic point of view, this implies that residues 168 to 176 of the C-terminal tail are also largely preordered, ready to accommodate the mineral surface. At first glance, the most dramatic interaction at the protein/mineral interface is mediated by the Lys171 side chain, which projects directly into the mineral. Presumably the positive charge of the ε-amino group serves as an iron mimetic to interact with the oxyanions in the mineral (Figure 5). And similar to the nucleated structure, the negatively charged Glu174 side chain and the main chain carbonyl groups of Pro170, Gly173 and Pro176 appear to provide additional interactions with the mineral surface.

**Figure 5:**
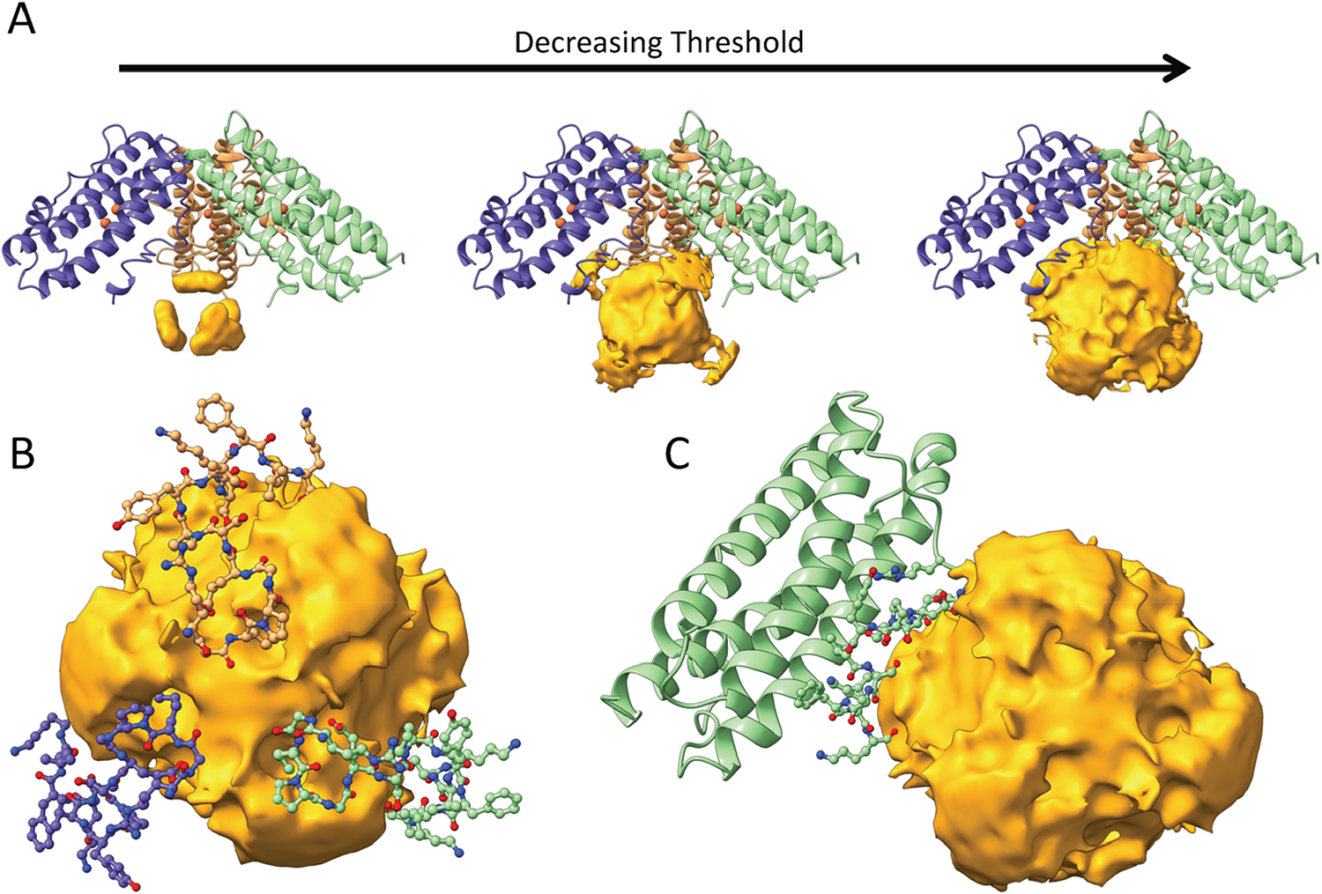
Iron mineral core formation in Pf-DPSL. (A) The difference density map (total map density – protein density) indicates the presence of mineralized Fe­(O)­OH in the tetrahedrally averaged iron loaded structure. When contoured at higher levels (left panel), density is localized to 4 small areas on the inner surface of the particle, each immediately surrounding the 3-fold axes. As the contour level is decreased (center and right panels), the density grows into the center of the particle, and out toward the 2-fold axes. (B) The interaction of residues 170–176 in the C-terminal tails (ball and stick) with the mineral core. Relative to panel A, the view is rotated 90 ° about the horizonal axis, looking down the 3-fold axis. (C) Relative to panel A, the view is a 120 ° rotation about the vertical axis, showing the interaction of the C-terminal tail of subunit B with the mineral core. Main chain carbonyl groups and acidic side chains are implicated in the interaction.

Interestingly, however, at higher contour levels the strongest density is found on the inner surface of the particle, adjacent to residues 168 to 171. These residues are proximal to the 3-fold axes, resulting in a 3-fold symmetric constellation on the interior surface of the nanoparticle beneath the ferritin-like pores. These residues also represent the last constriction point along the 3-fold pore where they appear to delineate the nucleation crucible or antechamber from the interior of the particle. Beginning at the highest contour level in the density map, as the contour level is reduced, the iso-surface then grows out from each of these clusters; laterally along the inner surface of the particle as well as into the center of the cavity (Figure 5A). This suggests mineral growth emanates from these 3-fold symmetric surfaces on the interior surface of the particle. Further, if the mineral is indeed present as ferrihydrite, it also suggests a 3-fold axis in ferrihydrite (space group *P*6_3_mc) should align coincident with a 3-fold axis in Pf-DPSL; and that if particles with a single crystallite could be identified, a C3 reconstruction might indeed resolve the mineral structure.

### Particle Sorting and C3 Reconstruction

To test this hypothesis, and because the 3-fold pores in DPSL are related to each other by D2 symmetry, the iron loaded data set was D2 symmetry expanded (see methods, Figure S5). Masks covering the densest mineral features coordinated by residues 168–171 at each of the four positions were then constructed and used to sort for the presence of mineral at the first site (lying on the *z* axis), but the absence of mineral at the remaining three sites. This identified 167,240 particles with significant iron density at a single 3-fold. Homogenous refinement with 3-fold (C3) symmetry then gave a map with strong mineral density for the C-terminal coordinated mineral along the Z axis (Figure 6). Importantly, this map also lacked density at the remaining 3-folds, validating the sorting protocol.

**Figure 6:**
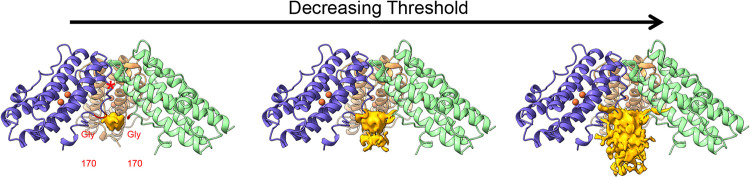
Difference density map (total map density – protein density) indicates the presence of ferrihydrite in the C3 averaged, mineralized structure. When contoured at higher levels (left panel), density is localized to the interior surface of the particle surrounding the 3-fold where it interacts with the main chain carbonyl group in Gly170. Notably, the antechamber (red asterisk) that housed the nucleated mineral now appears empty. As the contour level is reduced to intermediate (center) and lower (right) levels, the density grows laterally, as well as in toward to the center of the particle.

### Protein-Mineral Interactions II

While the overall resolution of this map was 2.5 Å, the mineral is significantly less ordered. Nevertheless, there was clearly resolved density for a constellation of 4 iron atoms with tetrahedral geometry centered on the 3-fold axis (Figure 7). The density is proximal to residues 168–171 and highly similar to the 4Fe/4O cluster in the ferrihydrite structure of Michel et al.^24^ Further, when the Michel ferrihydrite crystal structure was symmetry expanded and superpositioned upon the modeled irons, we identified an extended ferrihydrite superstructure with 13 Fe and 18 O atoms that was consistent with the surrounding density (Figure 7).

**Figure 7:**
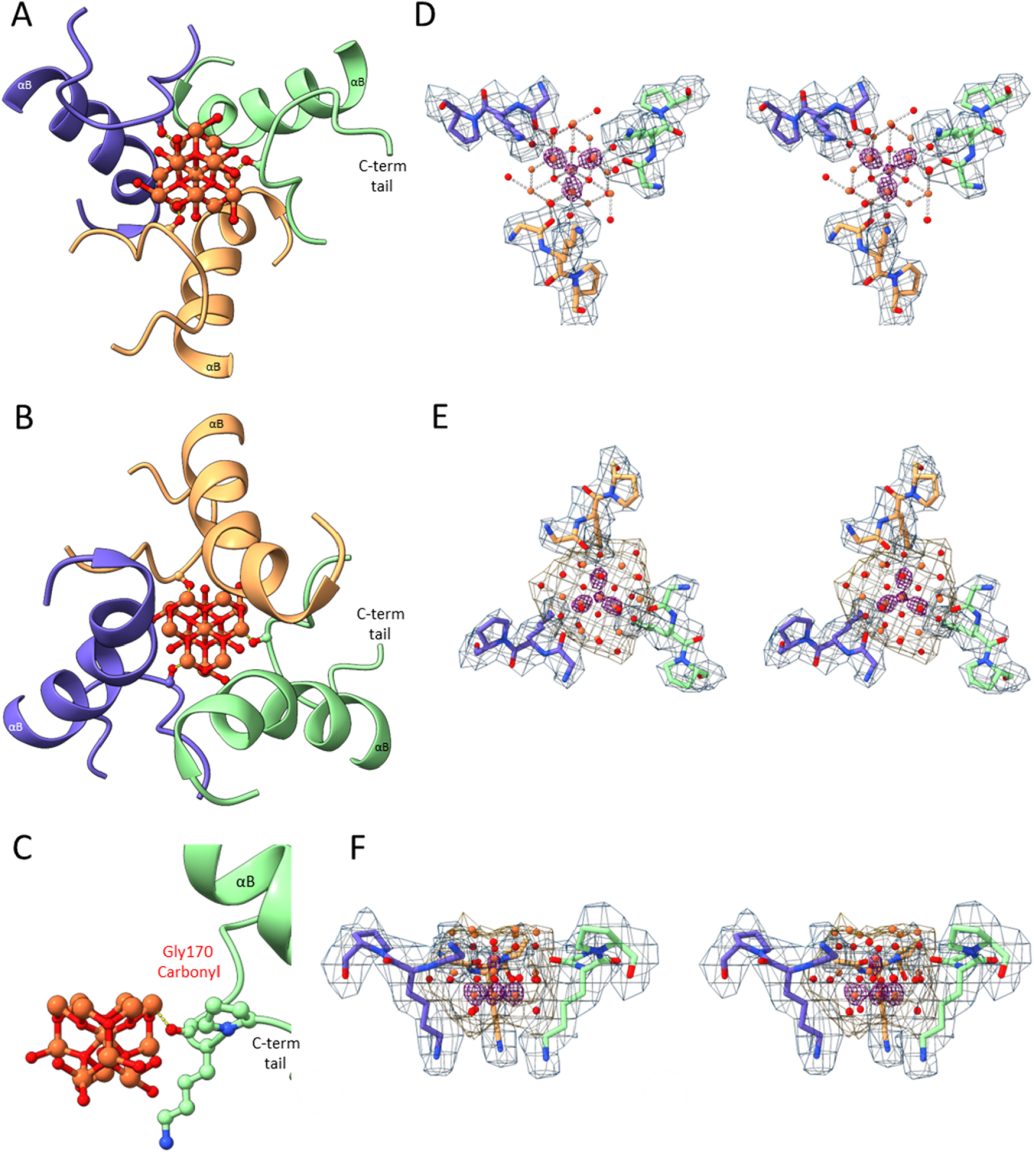
Ferrihydrite crystal growth on the inner surface of the acidic pore. (A) Looking along the 3-fold axis, from the inside of the particle out. The ferrihydrite structure of Michel et al. is depicted as spheres, with iron and oxygen atoms colored orange (rust) and red, respectively. The top portion of helix B along with residues 167 to 175 of the C-terminal tail are depicted as cartoons for subunits A, B and C (in blue, green and sandy brown). The Gly170 carbonyl groups are shown as ball and sticks, with the carbonyl oxygen in red. Dashed yellow lines indicate bonds between carbonyl oxygens and adjacent iron atoms. (B) The view is rotated 180 deg about the horizontal axis, now looking from the exterior of the particle toward the interior. (C) The view is now perpendicular to the 3-fold axis. Lys171 and Pro172 in the C-terminal tail are depicted in ball and stick. In this orientation, the ferrihydrite structure is “upside down” relative to the structure in panel 4C. The antechamber above the metal cluster is now empty in this structure. (D) Stereo view corresponding to the view in panel A. Potential density for the mineral is contoured at two different levels, 12.6 σ (magenta) and 4.5 σ (beige). Protein density (Dodger blue) is also contoured at 4.5 σ. At 12.6 σ, 4 individual iron atoms are nicely resolved, while at 4.5 σ the isonet covers the entire ferrihydrite model. (E) The model and map depicted in panel D are now in the same orientation as panel B. (F) The model and map depicted in panels D and E is now in the same orientation as panel C.

The Michel ferrihydrite structure contains 3 iron atoms in the asymmetric unit, which we denote Fe1, Fe2 and Fe3. In this context, a primary interaction with DPSL is between Fe1 and the carbonyl groups of Arg169 and Gly170. The interaction with the Gly170 carbonyl is particularly strong with a bond distance of ∼2.0 Å. Three such interactions occur in this immediate area due to the proximity to the 3-fold axis. These three Fe1 atoms are further coordinated through additional interactions within the mineral involving 3 additional Fe1 positions, and their bridging oxyanions. This gives rise to a top ring of 6 Fe1 atoms surrounding the 3-fold axis on the interior surface of the particle, that then grows out toward the center of the cavity (Figures 6 and 7).

### A Dynamic Pore Structure

These iron loaded structures revealed one additional surprise. While there was strong mineral density in the particle interior, the maps generally lacked density for the irons coordinated by Leu47 and Glu50 that were originally seen in the nucleated structure. In addition, density for the Glu54 coordinated irons was only apparent at the lowest contour levels. Further, significant solvent space separates the C-terminal coordinated mineral from the Glu54 coordinated irons. Thus, while iron fills the nucleation antechamber at the earliest time points, in the later, equilibrium iron loaded structures, mineral appears to have moved out of the antechamber, and the dominant mineral feature is instead coordinated by the C-terminal tail (Figures 6 and 7). On its own, this observation is unlikely to indicate a specific directional movement. In one scenario, and consistent with Ostwald ripening,^56–58^ nascent mineral might release from the nucleation site and move into the particle, contributing to crystal growth. Alternatively, it might instead point to a first in, first out scenario, in which the solvent exposed nucleation sites are involved in initial iron release to the exterior bulk solvent.

### The Ferroxidase Center

The iron loaded structure also shows significant change around the ferroxidase center (Figure 8). Density is still strong for the irons and their coordinating side chains, but instead of the bridging μ-oxo density between the irons that was present in earlier maps, cigar shaped density for a potential peroxo species is now present, with one oxygen coordinated to the A and B site irons, and the second to a water (HOH 200) in the solvent channel (Figure 8). In addition, there are several breaks in the main chain density along the solvent channel leading from the exterior surface to the ferroxidase center, indicative of a dynamic FOC. Despite this, density for the cysteine side chains of the thioferritin motif remains clear. The residues are reduced, with Cys118 present in dual conformations. In one, the side chain is now inserted into the channel leading to the di-iron site, where it also coordinates water HOH 200 (Figure 8). The second is in the “open” conformation seen in the unloaded and nucleated structures, that potentially allows substrates and products to move between the ferroxidase center and the exterior surface of the particle. This is the first observation of a peroxo species at the bacterioferritin-like ferroxidase center in DPSL, and the first time Cys94 has been seen protruding into the channel to interact with ligand via HOH200) at ferroxidase center.

**Figure 8:**
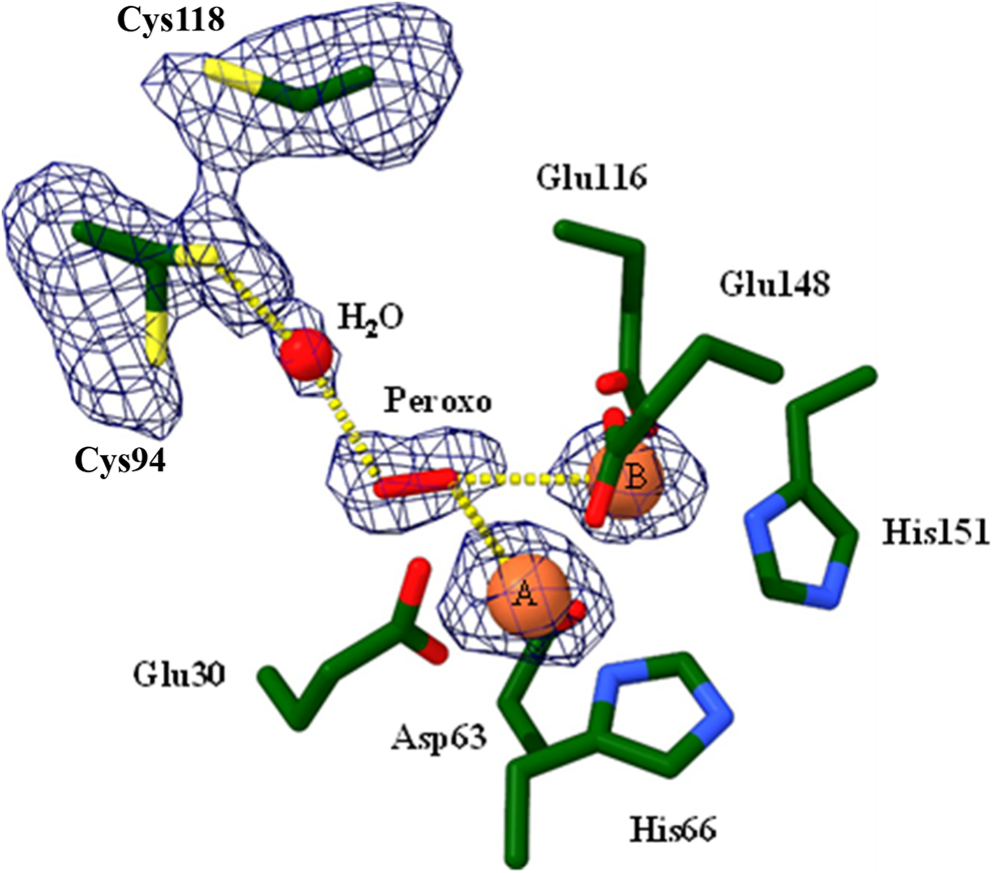
Density for the cysteine residues and features in the solvent channel leading to the ferroxidase center. Cys94 is present in two conformations. In one, the side chain reaches into ^the solvent channel to coordinate H^2^O 200. This central water is also coordinated to density^ consistent with a peroxo species, which is in turn coordinated to irons A and B.

## Discussion

### Nucleation

A multitude of high-resolution ferritin, bacterioferritin and DPS structures show iron density at the di-iron ferroxidase center. Crystallographic studies have also resolved locally ordered iron binding sites^31,59–62^ as well as 3- and 4- iron metalloclusters^19,33^ on the interior surfaces of several ferritins and DPS that have been suggested as possible sites of nucleation. And while a few Cryo-EM studies have resolved bulk mineral at low resolution,^31^ high-resolution structures that resolve nascent ferric oxyhydroxide mineral have been elusive. Indeed, to our knowledge the nucleated mineral structure described here represents the first such 3-dimensional mineral structure for any member of the ferritin superfamily. This structure implicates the symmetry match between the acidic pore and the iron oxyhydroxide as a key structural feature in guiding nucleation in Pf-DPSL. Further, in addition to the namesake glutamate residues, prepositioned main chain carbonyl groups at the acidic 3-fold also make very significant interactions with the surface of the nucleated mineral, not just to iron, but select oxygens as well, which are presumably present as hydroxyls.

The acidic 3-fold pore is a conserved feature of the mini-ferritins, present not only in DPSL,^18,28^ but DPS as well.^3,10,14,63^ In DPS, the acidic 3-fold pore is also referred to as the “ferritin-like” pore. And indeed, similar 3-fold pores are found in both ferritin and bacterioferritin. It is thus attractive to consider this may be a general feature of the mini-ferritins, and perhaps the ferritins in general. However, in DPS and ferritin, the hydrophilic 3-fold pores are also clearly implicated in iron entry.^64–70^ Whether the acidic 3-fold pore serves in both capacities in DPS or DPSL remains an open question.

### Catalysis of Nucleation

Classical nucleation theory considers the contribution of (i) volume dependent and (ii) surface area dependent free energy terms to the critical free energy of nucleation (ΔG*).^71,72^ The volume dependent term contributes favorably (–ΔG*), and is proportional to the radius of the nucleation embryo cubed (*r* ^3^). In contrast, surface area free energies are unfavorable and scale with the radius squared (*r* ^2^). At low radii, the surface area term dominates, inhibiting nucleation, but as the nascent embryo grows, the volume dependent term begins to dominate, resulting in a stable nucleus. The crossover between these competing terms defines the critical radius of nucleation (r*), and the energy at r* defines the activation energy for both homogeneous and heterogeneous nucleation. In heterogeneous nucleation, however, favorable interactions of the embryo with a “foreign” preformed surface reduce the surface area energy term; and in turn, the activation energy as well. Consistent with this classical nucleation theory, Pf-DPSL does indeed provide a preformed surface, rich in precisely positioned carbonyl and carboxylate groups that make energetically favorable interactions with the surface of the embryonic ferric oxyhydroxide. The resulting reduction in the surface area free energy, in-turn, results in a concomitantly reduced activation energy. Thus, by definition, Pf-DPSL efficiently catalyzes the nucleation of ferric oxyhydroxide by this mechanism. In this context, the structure of the 50-iron nucleated form contributes significant insight into the mechanism of catalytic nucleation.

### The C-terminal Tail and Mineral Growth

Using focused 3D classification, we were also able to resolve two structures of iron loaded Pf-DPSL. The first utilized iron loaded particles with significant mineral density in the center of the particle, while the second utilized a smaller subset of the particles with mineral density present on the interior surface surrounding a single 3-fold axis. The overall structure of the protein was nearly identical in each case, with each structure indicating a major role for the C-terminal tail (residues 170-176). While the C-terminal tail is preordered in Pf-DPSL, this is in direct contrast to Ss-DPSL, where the C-terminal tail is disordered, and Bf-DPSL where the C-terminal tail lies on the exterior of the particle. Clearly then, a preordered C-terminal tail on the interior surface of the particle is not a general feature of even the DPSLs.

However, the use of a checkerboard pattern of positive and negative charge, along with heavy reliance on strategically placed carbonyl groups is likely a common mechanistic feature for the biomineralization of iron across the ferritin superfamily. Further, because the Pf-DPSL C-terminal tail does not extend into the interior of the iron mineral, but is instead found at the protein/mineral interface, it is attractive to consider that interior tails of other ferritin superfamily members will also adopt conformations at the protein mineral interface, rather than inserting deeply into the mineral phase. From this point of view, disordered internal tails in Ss-DPSL and other members of the ferritin superfamily may indicate the carbonyl groups in these tails are indeed unsaturated, and thus available to interact with mineral.

### Structure of the Mineralized Core

Ferrihydrite is a fine grained, poorly crystalline nanomineral that is widely distributed across freshwater and marine systems, aquifers and soils; where it plays an important role in the geochemical cycling of iron as a precursor to the more stable iron oxides.^73^ It is also thought to be the dominant iron oxide phase on the surface of Mars, responsible for its red hue, and thus attesting to a wet period on early Mars.^74^ Regardless, due to its low cost, small grain size, large and highly adsorbent surface area, it is frequently used on earth for large scale water treatment to remove unwanted metals and organic components. These same properties, however, have complicated structural studies, and despite a half century of effort, we still lack a unanimously accepted model for ferrihydrite. Currently, two competing models are considered in the literature.^23,24^ Notably, in our second iron loaded structure, with mineral density present at one and only one 3-fold pore, we were able to resolve mineral density in the immediate vicinity of a single 3-fold axis. Based upon our modeled iron atom positions, we found the density to be consistent with the Michel et al. ferrihydrite structure.^24^ Importantly, however, individual oxygen atoms are not well resolved in this map, and their positions, while consistent with the density, are inferred from the superpositioned Michel model. In this context, the evidence for tetrahedral oxygen coordination for the third iron (Fe3) in the Michel asymmetric unit is indirect.

Interestingly, a recent *ab initio* thermodynamics study by Sassi et al. suggests the Michel and Manceau models are thermodynamically equivalent over a wide range of temperature and pressure conditions, with higher temperatures and water pressures favoring the Michel model, and lower temperatures and water pressure favoring the Manceau model.^25^ In this context, we note that *P. furiosus* is an extremophile with an optimal growth temperature of 100 °C (373 K), and that a Michel-like structure might thus be expected. We wonder whether a psychrophilic, or even mesophilic ferritin might instead nucleate or crystallize a Manceau-like structure? Subtle remodeling at the ferritin-like 3-fold pore might allow it to template the nucleation of other 3-fold symmetric minerals, including the Manceau et al. ferrihydrite model.^23^ In any event, observation of a Michel-like structure does not necessarily preclude the existence of the Manceau model.

### Biomineralization

Overall, the 3-fold symmetry of Pf-DPSL functions to template both nucleation and crystallization of iron oxyhydroxide. In addition, main chain carbonyl groups are found to play a critical role in both processes. At the earliest time points, ordered iron oxyhydroxide is clear in the antechamber of the 3-fold pores. At later time points, greater mineral density (higher occupancy) is present on the inner surface of the particle, immediately surrounding the 3-fold axes, where we observe a locally crystalline matrix. In addition, particle sorting indicates ongoing biomineralization at two or more symmetry related sites (on average) within a single particle. However, because there are four such sites per particle, not all are fully occupied. This suggests that once crystallization begins at one or more sites, the nascent crystallites become the dominant sites for crystal growth, with crystalline mineral then growing from these sites, both laterally and toward the center of the particle, until eventually colliding with mineral growth from a symmetry related site, resulting in the formation of intraparticle grain boundaries.

In addition, after the reaction reaches equilibrium, we see decreased mineral density at the nucleation sites. This might indicate movement into the stable crystalline core, which could be thermodynamically favored (Ostwald ripening).^56–58^ Notably, such movement is consistent with the model for human hepatic ferritin proposed by Pan et al., in which early iron oxyhydroxide deposits are proposed to diffuse inward, contributing to the formation of the local crystalline arrays observed in micrographs of cytosolic human ferritin from biopsies.^31^

In the iron loaded structures, we also see evidence for a dynamic situation at the ferroxidase center. However, a potential path from the ferroxidase center that would allow oxidized iron to enter the core is not obvious in any of the Pf-DPSL structures. On the contrary, as mineral begins to accumulate, the tryptophan (Trp154, Figure S9) juxtaposed between the 3-fold pore and the ferroxidase center becomes ordered, which along with the ordered C-terminal tail, would seem to restrict Fe­(II) movement into the cavity. This might in turn suggest the major path for iron entry is instead through the remaining acidic 3-fold pores. This model is largely similar to the “linked transfer” model for transferrin and bacterioferritin,^67^ in which Fe­(II) enters through the pore and is oxidized to Fe­(III) within the particle cavity. The resulting electrons then flow from the core to the ferroxidase center, where they reduce O_2_ to H_2_O_2_, or H_2_O_2_ to water (Figure 8). At the same time, water in the core is sequentially deprotonated from H_2_O to OH^–^, and OH^–^ to O^2–^, as it is incorporated within the biomineral core. Alternatively, ferroxidase activity that reduces peroxide to water may be independent of the mineralization activity. Importantly, the ability to follow biomineralization of Pf-DPSL by cryo-EM may enable future structure–function studies to elucidate these important mechanistic details.

## Supplementary Material



## Notes

Exemplar micrographs, cryo-EM workflows, representative potential density for the unloaded, nucleated and iron loaded structures, supporting figures for the unloaded, nucleated and iron loaded structures (PDF)
