# Supplementary material for: The Mechanism of Mineral Nucleation and Growth in a Mini-Ferritin
Source: J Am Chem Soc. 2025 Oct 6;147(41):37030–44. doi: 10.1021/jacs.5c05464 (PMC12532284; doi:10.1021/jacs.5c05464)
Supplement: Supplementary file 1 [file ja5c05464_si_001.pdf]

Supplemental Information

The Mechanism of Mineral Nucleation and Growth in a Mini-Ferritin

Colin C. Gauvin[a,b], Monika Tokmina-Lukaszewska[a], Hitesh Kumar Waghwani[c], Sterling C. McBee[a], Trevor Douglas[c], Brian Bothner[a] and C. Martin Lawrence[a,b,*]

[a]Department of Chemistry and Biochemistry Montana State University, Bozeman, MT, 59717, USA

[b]The Thermal Biology Institute, Montana State University, Bozeman, MT, 59717, USA

[c]Department of Chemistry, Indiana University, Bloomington, Indiana 47405, USA

SUPPLEMENTAL METHODS

Expression and purification of Pf-DPSL

E. coli BL21 cells were transformed with 5 ng of vector, and plated onto agar plates with 30 mg/mL kanamycin, and grown overnight at 37 °C. A fresh colony was picked and used to inoculate 5 mL of lysogeny broth (LB) media (1% tryptone w/v, 0.5% yeast extract w/v, 0.5% NaCl w/v), in a 15 mL plastic centrifuge tube with the lid loosened, in a shaking 37 °C incubator for 12 hours. 750 µL of the overnight culture was used to inoculate 750 mL cultures of LB media with 30 mg/mL kanamycin, which were grown in 3.5 L baffled flasks at 37 °C for 18 hours, with no induction (leaky expression). The cells were harvested via centrifugation in a Beckman Model J-6B centrifuge using a JS-5.2 swinging bucket rotor at 3000 RCF for 30 minutes. The pellets were stored at -20 °C until subsequent purification.

As needed, pellets containing expressed *Pf*-DPSL were thawed, resuspended in 100 mM NaCl and 50 mM MES at pH 6.5 with, at a ratio of 5 mL buffer per 1 g of cell pellet, and then lysed in a French press. The lysate was immediately spun at 30,000 RCF for 45 minutes in a Sorvall RC-2B equipped with a Fiberlite F21 fixed angle rotor. The soluble fraction was then heated at 85 °C for 10 minutes, before being spun again. After centrifugation, the cleared lysate was divided into 1 mL aliquots and frozen at -80 °C. When needed, aliquots were thawed, filtered with a 0.22 µm PVDF filter, and passed over a Superose 6 size exclusion column equilibrated with 100 mM NaCl in 50 mM MOPS at pH 6.5. DPSL eluted at a volume of 14.5 mL, corresponding to a molecular weight of approximately 280 kDa. Purified DPSL was concentrated to 2.5 mg/mL (measured via Bradford assay[34]), before being frozen again at -80 °C. For all downstream assays, *Pf*-DPSL aliquots were thawed and centrifuged at 16,000 RCF for 5 minutes in a microcentrifuge, and the supernatant was drawn off for subsequent use.

Strategies for Single Particle Analysis of Iron Loaded Ferritins

Notably, *ab initio* alignment and refinement of iron-loaded particles was difficult, likely due to the high amplitude contrast of the iron core, and heterogeneity in the size, shape and location of the mineral within the particles. Even the largest datasets routinely failed to give high resolution details for the protein main chain. Instead, alignments seemed to over-weight the mineral core. This problem could be mitigated in a variety of ways, although not without some tradeoffs. In a mixed particle dataset with roughly equal proportions of empty and mineralized particles, the two species refined nicely together to high resolution. At that point, particles could be classified in either 2D or 3D, and the mineral-containing particles alone could be reconstructed using the poses from the original mixed refinement, giving maps to intermediate or high resolution. Alternatively, the mineral core could be subtracted prior to refinement, although strong residual density near the 2-fold axes still lead to spurious alignments in some cases. Subtracted particles, once refined, can then be re-extracted with mineral, and reconstructed using the refined particle poses from the particle subtracted structure. However, this typically resulted in lower-than expected resolution, relative to reconstruction with an identical number of empty particles. In any event, and perhaps contrary to expectations, an initial reconstruction using a heterogenous mix of both empty and full particles gave better starting poses for the iron containing particles, which could then be selected and taken forward for the iron loaded structures. We believe inclusion of empty particles indirectly focuses alignment on the protein shell, as it is the common feature across all particles, whereas *ab initio* solutions using only the

iron containing particles are dominated by the high amplitude, heterogenous features of the mineralized iron core.

Native Mass Spectrometry

Iron loading was investigated using native mass spectrometry. The experiments were conducted on a SYNAPT G2-*Si* instrument (Waters) as described previously.[49,50] Briefly, the samples were washed with 100 mM ammonium acetate solution, pH 6.5 (Sigma) using 30-kDa molecular weight cutoff spin filters (Pall Corporation) and then immediately infused from in-house prepared gold-coated borosilicate glass capillaries to the electrospray source at a protein concentration of 4 μM. The instrument was tuned to enhance performance in the high mass-to-charge range (positive mode) with the following settings: source temperature 30 °C, capillary voltage 1.1-1.4 kV, trap bias voltage 16 V and argon flow in collision cell (trap) 7 mL/min. Transfer collision energy was held at 5 eV while trap energy varied between 5 and 100 eV. Data analysis was performed in MassLynx software version 4.1 (Waters). The observed signal for unloaded DPSL was on average higher by 1386.3 m/z than that expected for 12 copies of the apo protomer, potentially corresponding to a heterogenous mixture of Fe and Zn at the ferroxidase centers. While 24 irons might be expected, the m/z values for unloaded DPSL are best fit by 12 Fe and 11 Zn per dodecamer. In addition, also present in the mass spectra are two minor species of +1195.5 m/z (12Fe/8Zn) and + 1637.5 m/z (13Fe/14Zn).

SUPPLEMENTAL FIGURES

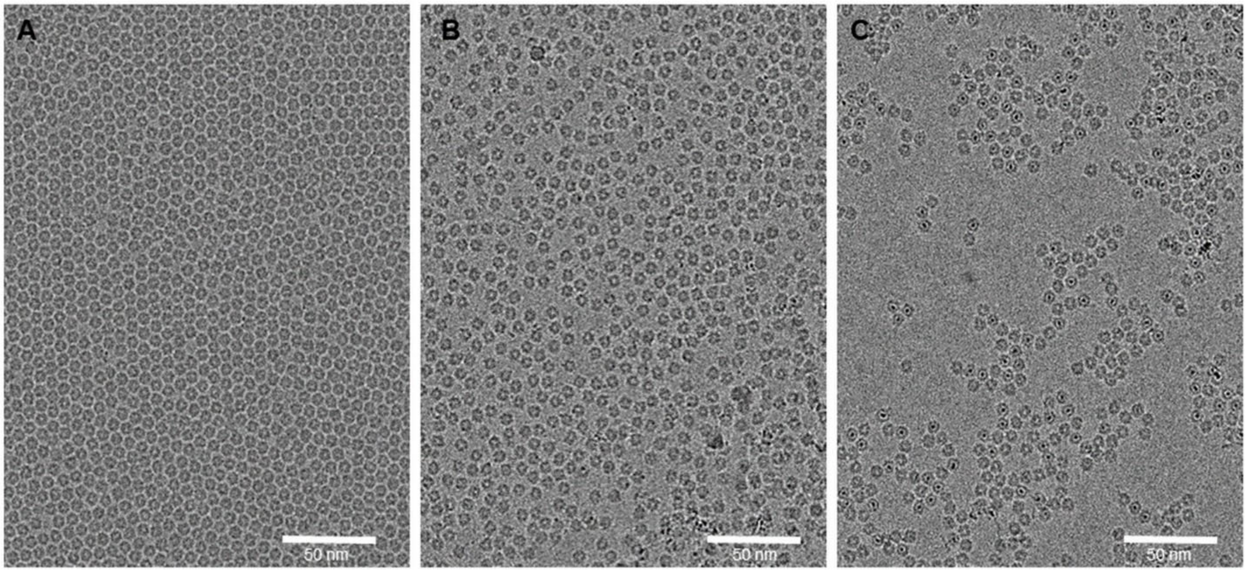

Figure S1. Representative micrographs from the empty (A), nucleated (B), and mineralized (C) datasets. The mineralized data set shows clear heterogeneity with respect to iron loading, with black punctate points in the center of many particles that indicate significant mineralization, while others that appear largely empty. Each micrograph was collected at 88,000 x magnification with a total dose of ~55 e/Å^2 and -1.4 μm defocus. Scale bars are 50 nm.

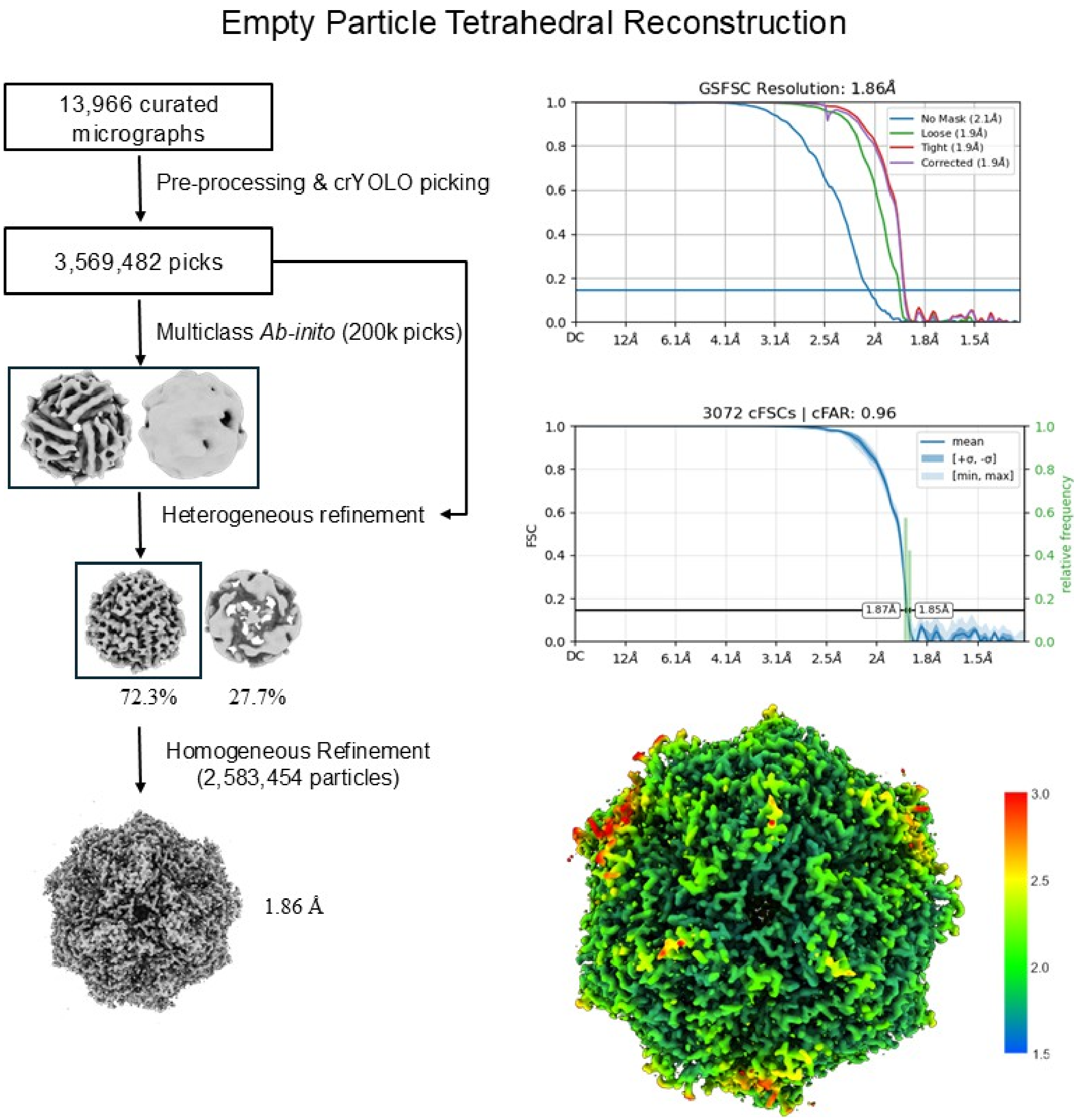

Figure S2. Empty *Pf*-DPSL single particle analysis workflow. Please see methods for details. The Gold Standard FSC output from CryoSPARC is shown in the upper right panel. The 3D or conical FSC is shown in the middle right panel, with an exceptional cFAR value of 0.96 indicating the robust distribution of particle orientations. Note that in the absence of iron loading, the standard deviation, min and max correlation values are tightly grouped about the mean.

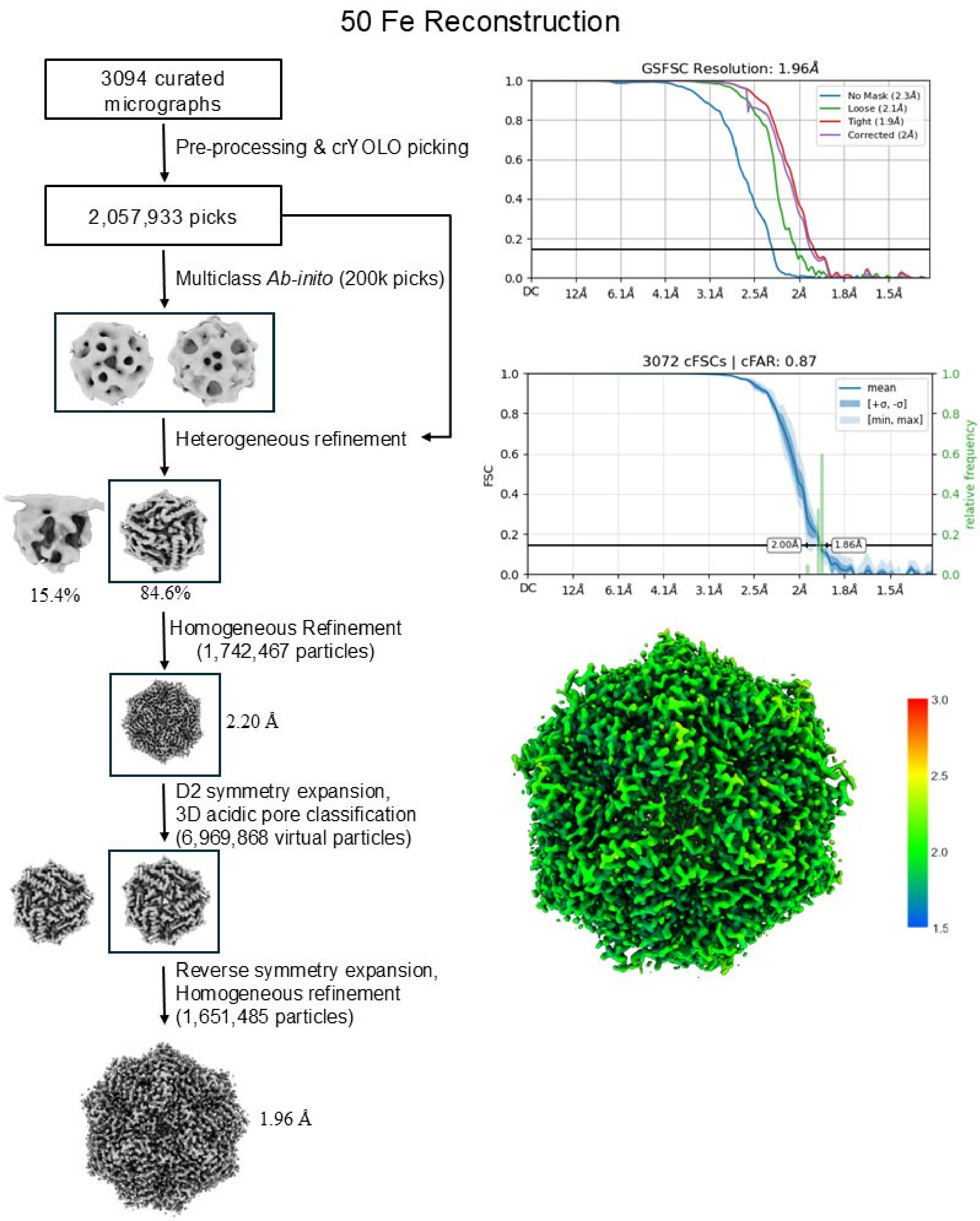

Figure S3. Nucleated *Pf*-DPSL single particle analysis workflow. Please see methods for details. The Gold Standard FSC output from CryoSPARC is shown in the upper right panel. The 3D or conical FSC is shown in the middle right panel, with a cFAR value of 0.87, indicating a robust distribution of particle orientations. In the case of limited iron loading, the standard deviation, minimum and maximum correlation values are again tightly grouped about the mean.

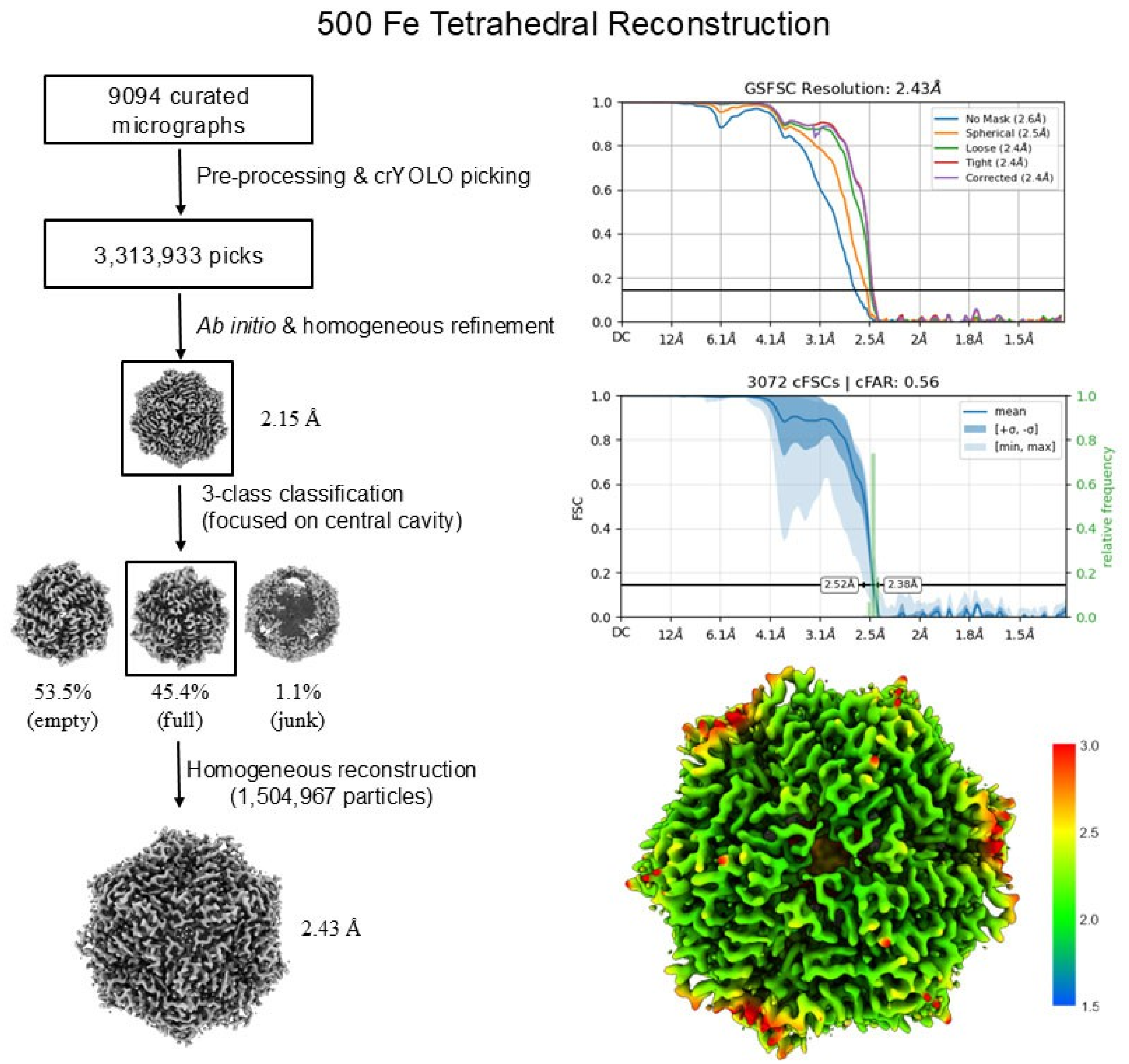

Figure S4. Mineralized *Pf*-DPSL single particle analysis workflow. Please see methods for details. The Gold Standard FSC output from CryoSPARC is shown in the upper right panel (2.43 Å). The 3D or conical FSC is shown in the middle right panel, with a cFAR value of 0.56. While indicating some directional anisotropy, CryoSPARC documentation suggests a cFAR value above 0.5 serves as a reasonable threshold for the absence of a significant preferred orientation distribution. The cFSC plot also shows significant variation from the mean between 3 and 4 Å, that disappears at higher spatial frequencies, likely due to the presence of the poorly ordered mineral as it grows into the interior of the capsid, demonstrating the challenge of working with iron loaded particles.

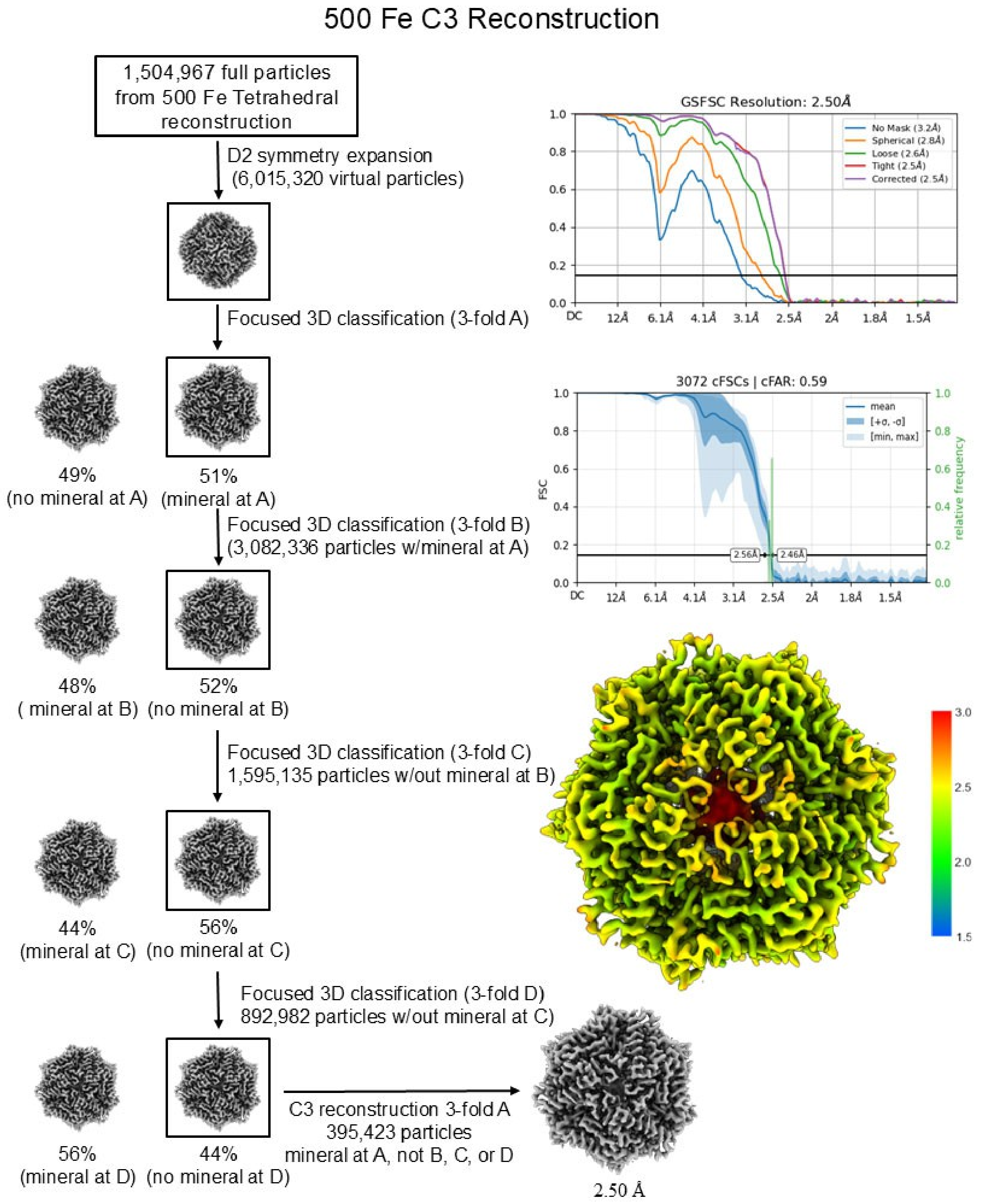

Figure S5. Mineralized C3 single particle workflow. Please see methods for details. The Gold Standard FSC output from CryoSPARC is shown in the upper right panel (2.5 Å). The 3D or conical FSC is shown in the middle right panel, with a cFAR value of 0.59. Interestingly, we observed a slight increase in cFAR despite using only a small subset of the particles that went into the tetrahedral reconstruction. While this might at first be contrary to expectation, it may indicate an improvement based on the selection of particles with lower iron content. Regardless, while the cFAR indicates some directional anisotropy, CryoSPARC documentation suggests cFAR value above 0.5 serve as a reasonable threshold for the absence of a significant preferred

orientation distribution. As for the tetrahedral reconstruction, the cFSC plot also shows significant variation from the mean between 3 and 4 Å, that disappears at higher spatial frequencies, likely due to the presence of the poorly ordered mineral as it grows into the interior of the capsid, further demonstrating the challenge of working with the iron loaded particles.

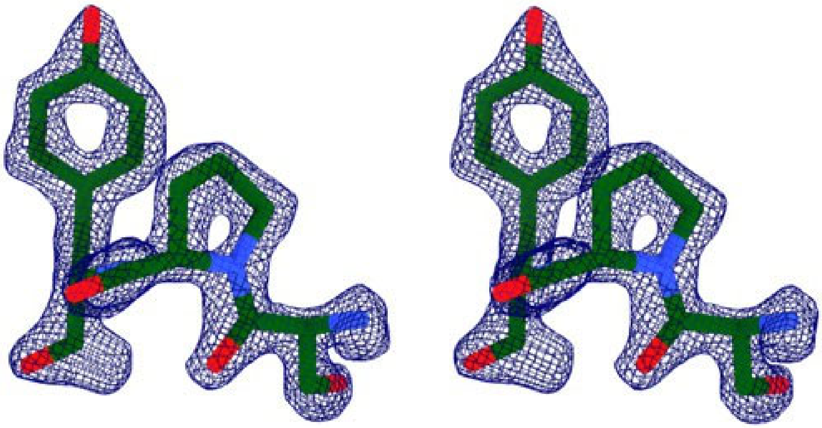

Figure S6. Stereo image of representative potential density for the empty structure. Shown are Ser^{175}-Pro^{176}-Tyr^{177}.

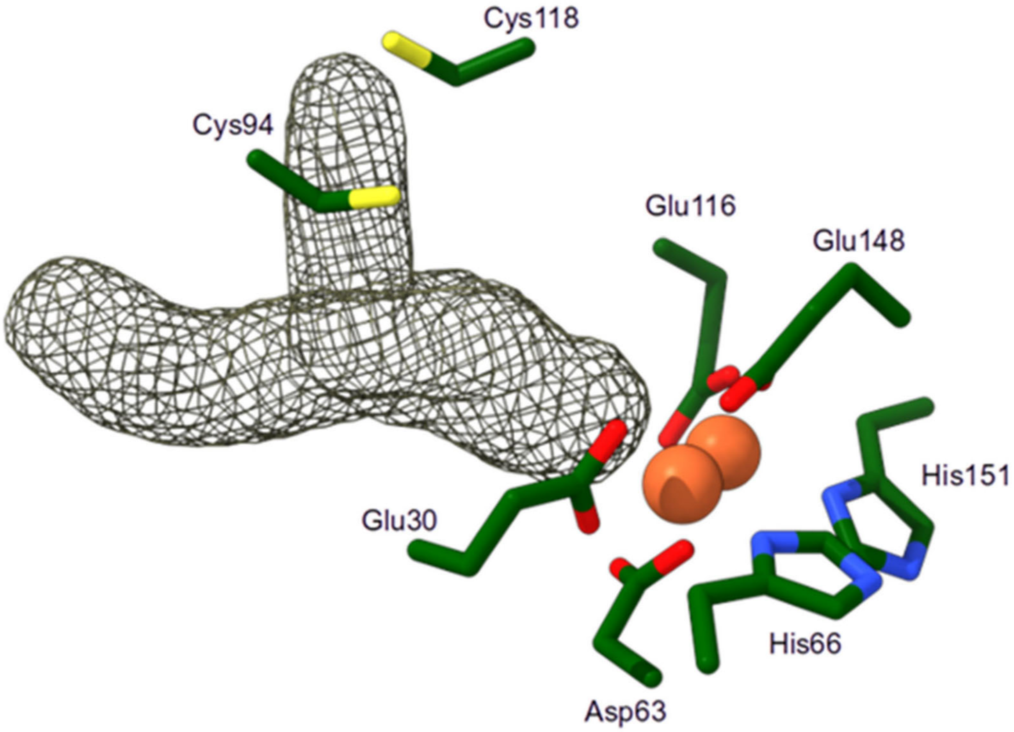

Figure S7. Conserved side chains in the *Pf*-DPSL bacterioferritin-like ferroxidase center, including the conserved cysteine pair, are shown in sticks. The 2 irons in the ferroxidase center are rust colored spheres. And the solvent accessible channels leading into the FOC are represented by the brown net.

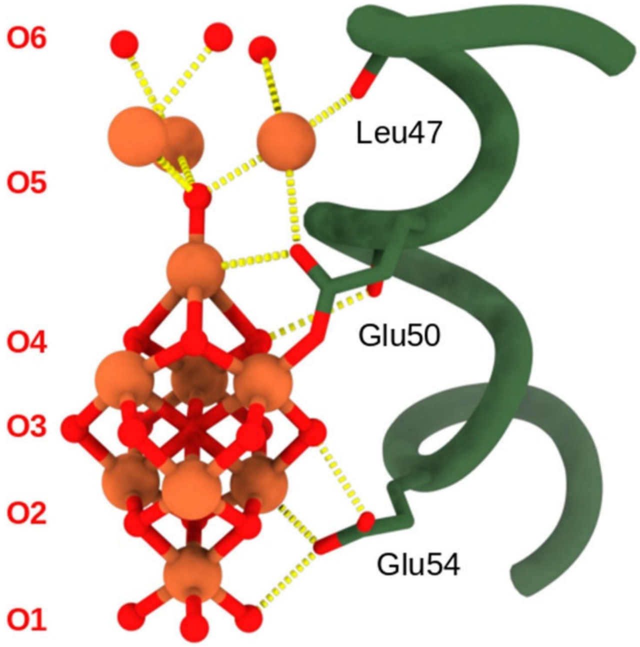

Figure S8. The nucleated structure can be described as alternating horizontal planes of close packed oxygen and interstitial iron atoms. Six oxygen planes, denoted O1 through O6 are present. A single *Pf*-DPSL subunit makes 8 contacts to the nascent mineral, giving 24 contacts per acidic 3-fold pore that participate in catalyzing mineral nucleation. Layers O1 to O4 are cubic close packed (ABCA), while layers O3 to O6 (ACAC) are hexagonal close packed, with layers O3 and O4 corresponding to the stacking fault between the cubic and hexagonal close packed structures.

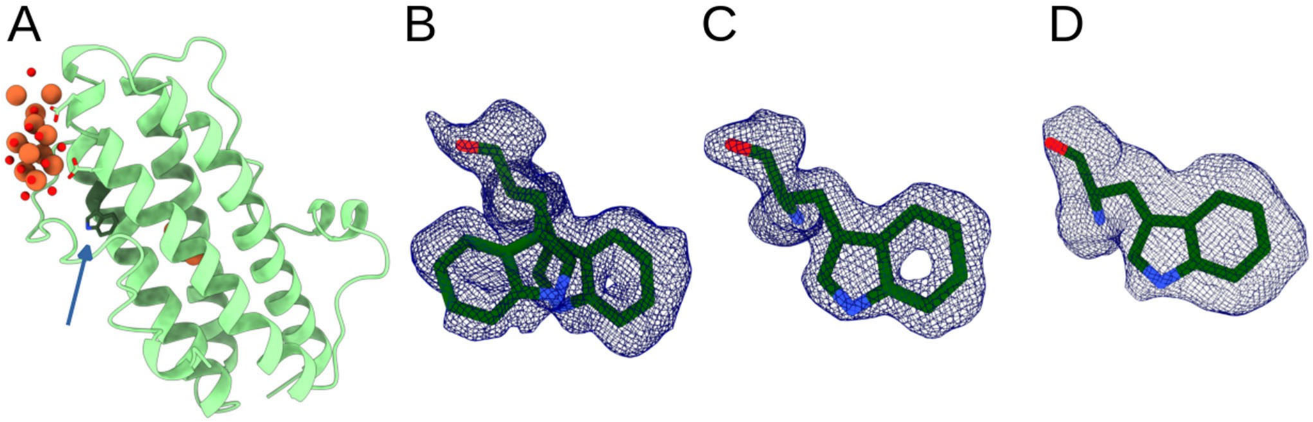

Figure S9. A) The location of Trp-154 relative to the site of mineral nucleation is shown for a single subunit. Notably, it lies intermediate between the nucleation site and the ferroxidase center buried within the middle of the 4-helix bundle. B) Trp-154 density from apo *Pf*-DPSL, depicting alternate locations A and B. Global map resolution 1.86 Å. C) Trp-154 density in the nucleated *Pf*-DPSL, with global map of resolution 1.91 Å, now indicates a single, well-ordered conformation for this residue. D) Trp-154 density from mineral loaded *Pf*-DPSL. The tryptophan remains in the single ordered conformation. Global map resolution 2.43 Å.

Table S1. Cryo-EM data collection, processing, refinement and validation.

	Unloaded	Nucleated	Iron Loaded (3-fold)	Iron Loaded
**PDB ID**	9CZ0	9CZ8	9E8S	9CZ9
**EMDB ID**	EMD-46055	EMD-46063	EMD-47728	EMD-46064
**Data Collection/Processing**				
Magnification	72,463x	72,463x	72,463x	72,463x
Voltage (kV)	200	200	200	200
Electron exposure ($e^-/Å^2$)	55	55	55	55
Defocus range (μm)	-0.6 -to -1.5	-0.6 to -1.5	-0.6 to -1.5	-0.6 to -1.5
Pixel Size	0.345 (super-resolution)	0.690	0.690	0.690
Initial number particles	3,569,482	2,057,923	3,313,933	3,313,933
Final number particles	2,583,454	1,619,844	395,423	1,504,967
Symmetry imposed	T (23)	T (23)	C3	T (23)
**Composition (#)**				
Chains	12	16	13	12
Atoms	17,040	17188	16831	17016
Residues	Protein: 2040	Protein: 2040	Protein: 2040	Protein: 2040
Water	336	516	144	540
Ligands	FE: 24	FE: 68	FE: 37	FE: 24
		O: 68	O:18	
			Peroxo :12	
**Bonds (RMSD)**				
Length (Å) (# > 4σ)	0.009 (0)	0.009 (0)	0.005 (0)	0.006 (0)
Angles (°) (# > 4σ)	1.033 (0)	0.800 (0)	0.969 (0)	1.16 (0)
MolProbity score	0.98	1.29	1.41	1.46
Clash score	2.06	5.32	7.51	8.47
**Ramachandran (%)**				
Outliers	0	0	0	0
Allowed	1.19	1.19	0.25	1.19
Favored	98.81	98.81	99.75	98.81
**Rama-Z (RMSD)**				
whole (N = 2028)	1.34 (0.18)	1.59 (0.17)	0.45 (0.18)	-2.22 (0.16)
helix (N = 1452)	0.99 (0.13)	1.16 (0.12)	0.07 (0.12)	-1.75 (0.11)
sheet (N = 0)	--- (---)	--- (---)	--- (---)	--- (---)
loop (N = 576)	0.97 (0.28)	1.14 (0.26)	1.41 (0.29)	-0.13 (0.25)
**Outliers**				
Rotamer outliers (%)	0	0.0	0.23	0
Cβ outliers (%)	NA	NA	0.36	NA
Cis proline/general	0.0/0.0	0.0/0.0	0.0/0.0	0.0/0.0
CaBLAM outliers (%)	0.6	1.2	0.00	0.6
**ADP (B-factors)**				
Iso/Aniso (#)	17,040/0	17,188/0	16831/0	17,112/0
min/max/mean				
Protein	2.98/75.27/21.33	8.25/60.3/22.53	4.21/79.88/33.72	0.12/60.08/10.15
Nucleotide	---	---	---	---
Ligand	31.77/52.08/41.93	0.01/100.33/58.06	30.09/200.00/106.63	12.78/19.59/16.18
Water	1.51/33.76/14.06	24.77/52.36/34.20	6..41/33.79/23.48	7.29/45.01/18.39
**Occupancy**				
Mean	0.99	0.98	1.00	1.00
occ = 1 (%)	97.04	95.44	99.29	99.30
0 < occ < 1 (%)	2.96	4.54	0.71	0.70
occ > 1 (%)	0.00	0.00	0.00	0.00
**Box**				
Lengths (Å)	105.6, 109.0, 101.4	104.19, 110.4, 102.81	105.57, 113.16, 104.88	105.95, 112.47, 104.19
Angles (°)	90.0, 90.0, 90.0	90.0, 90.0, 90.0	90.0, 90.0, 90.0	90.0, 90.0, 90.0
**Resolution**				
Supplied Resolution (Å)	1.86	1.96	2.43	2.50
Resolution Estimates (Å)	Masked Unmasked	Masked Unmasked	Masked Unmasked	Masked Unmasked
d FSC (half maps; 0.143)	1.86 2.1	1.91 2.2	2.43 2.6	2.5 2.6
d 99 (full)	2.1 2.1	2.2 2.2	2.6 2.6	2.5 2.5
d model	2.1 2.1	2.2 2.2	2.6 2.6	2.5 2.5
d FSC model (0.5)	1.9 1.9	2.0 2.0	2.5 2.6	2.5 2.5
Map min/max/mean	-28.58/52.59/-0.01	-28.48/63.39/0.05	-0.59/0.90/0.01	-22.93/43.12/0.57
**Model vs. Data**				
CC (mask)	0.88	0.86	0.80	0.83
CC (box)	0.77	0.73	0.72	0.79
CC (peaks)	0.80	0.76	0.67	0.75
CC (volume)	0.86	0.84	0.76	0.81
Mean CC for ligands	0.69	0.61	0.70	0.71
